# Quadriceps force direction affects patellofemoral kinematics without impacting tibiofemoral stability: a cadaveric study

**DOI:** 10.1186/s43019-025-00286-1

**Published:** 2025-08-28

**Authors:** Vera Maioli, Michele Conconi, Emanuele Diquattro, Francesco Traina, Nicola Sancisi, Luca Cristofolini

**Affiliations:** 1 Department of Industrial Engineering, School of Engineering and Architecture, Alma Mater Studiorum-University of Bologna, Via Umberto Terracini 24-28, 40131 Bologna, Italy; 2 Orthopaedic-Traumatology and Prosthetic Surgery and Revisions of Hip and Knee Implants, IRCCS Rizzoli Orthopaedic Institute, Via Pupilli 1, 40136 Bologna, Italy

**Keywords:** Patellofemoral joint kinematics, Knee, Quadriceps, In vitro biomechanical testing, Tibiofemoral joint, Optoelectronic system

## Abstract

**Background:**

Surgical interventions to correct abnormal quadriceps direction are performed in cases of patellofemoral joint disorders, to medialize the patella and reduce lateral stress. However, excessive medialization can lead to increased contact forces and joint instability, underscoring the need for a more detailed understanding of the effects of quadriceps alterations on joint biomechanics. The purpose of this study was to evaluate the impact of variations of the magnitude and direction of the quadriceps force on the kinematics of the patellofemoral joint.

**Methods:**

A total of 12 cadaveric knees were evaluated in flexion–extension applying different loads to the quadriceps tendon. Specifically, we evaluated five different directions of the quadriceps line of action in the frontal plane: neutral, ±6° and ±12°; and two directions in the sagittal plane: neutral and 5° anterior. Three load magnitudes were simulated: 20 N, 160 N, and 280 N. Relative motion between the patella, femur, and tibia was measured using an optoelectronic system.

**Results:**

The comparison under reference loading conditions (neutral direction, 20 N) across all specimens demonstrated consistent patellofemoral motion. Similarly, tibiofemoral kinematics was comparable between specimens and with the literature. Variations of the direction of the quadriceps force in the frontal plane exerted a significant impact on all components of motion in the patellofemoral joint. Compared with the reference condition, at full extension, 12° medialization increased patellar varus rotation (−6.2° ± 3.3°), while at high flexion it increased valgus rotation (4.8° ± 4.8°). Lateralization reversed this pattern, causing valgus at extension (7.7° ± 3.6°) and varus in flexion (−2.8° ± 1.8°). Medial–lateral patellar translation exceeded ±6 mm under 12° deviations. Sagittal-plane changes had minimal impact, mostly in extension when the patella is not yet in the trochlea. Tibiofemoral kinematics was more sensitive to load magnitude, although frontal-plane direction also affected joint rotation.

**Conclusions:**

This study provides essential insights into the biomechanical interplay between quadriceps alignment and patellofemoral kinematics. These findings may inform surgical strategies for optimizing patellar tracking.

*Level of evidence* In vitro biomechanical tests.

**Supplementary Information:**

The online version contains supplementary material available at 10.1186/s43019-025-00286-1.

## Introduction

The quadriceps muscle plays a pivotal role in knee biomechanics by directly influencing patellar kinematics [1]. Any imbalance or weakness in the quadriceps, particularly during eccentric contractions, can lead to altered patellar tracking [2] and increased stress on the patellofemoral (PF) joint [3]. Clinical observations frequently associate hypotrophy or diminished activity in the vastus medialis with imbalances relative to the vastus lateralis, with localized stress concentrations in the PF joint [4, 5]. These alterations can impair knee function, contributing to anterior knee pain [6, 7], PF instability [5], and even PF arthritis, among the most common clinical disorders in the PF joint [8, 9]. These alterations may also produce alterations in the tibiofemoral (TF) kinematics [10], with additional effects on joint stability [11].

Understanding the role of the quadriceps muscle in patellar kinematics is essential in clinical practice when managing knee disorders, as it directly affects joint alignment, load distribution, and long-term joint health. The force exerted by the quadriceps muscle is described by the quadriceps vector (QV). It represents the resultant force direction of the four quadriceps muscles acting on the patella. Clinically the direction of the QV is simplified by the Q-angle, defined as the angle formed between the lines connecting the center of the patella to the attachment site of the patellar tendon on the tibial tubercle, and the line connecting the center of the patella to the anterior superior iliac spine on the pelvis, with the knee fully extended. Typically, the Q-angle ranges from 6° to 27°, with an average value around 15° [12]. Although the Q-angle is widely used in clinical practice, this (by definition) is more a description of bone morphology than the actual direction of the quadriceps force [12]. While bone morphology influences the QV, it does not fully determine its orientation.

Individuals with an abnormally wide Q-angle are very likely to experience knee pain [13] or patellar instability [5]. A large Q-angle is generally associated with a more lateral QV, but unlike the Q-angle, the QV direction can be altered through specific surgical interventions. Several surgical treatments, such as medial PF ligament reconstruction [14], patellar realignment [15] through medialization and anteriorization (Fulkerson osteotomy technique) [16], tibial tubercle medialization, distal femur osteotomy, or reshaping the femoral trochlea through trochleoplasty [17], aim to realign the patella, restoring dynamic patellar stability and correcting patellar tracking. These procedures may secondarily affect the quadriceps vector direction by reducing the Q-angle or shifting patellar alignment. In this context, procedures such as lateral retinaculum release [18], patellar medialization and anteriorization (e.g., Fulkerson osteotomy) [15, 16], and trochlear reshaping [17] do not aim to manipulate the QV directly but rather produce vector modifications as a biomechanical consequence of realignment.

However, even if it is not the primary goal, changing the direction of action of the quadriceps does in turn influence the tracking of the patella [19, 20]. A lateralized QV increases the lateral force component acting on the patella, worsening misalignment and potential joint wear [21]. Despite the shared goal of medializing the patella, excessive medialization has been reported to elevate PF contact forces, potentially causing new complications [22, 23].

Knowledge of how quadriceps load patterns influence PF kinematics remains incomplete, particularly in the context of three-dimensional motion analysis. Understanding the relationship between PF kinematics, TF joint and quadriceps loading would provide crucial information not only for the optimization of strategies in case of PF complications, such as instability, pain, or maltracking, but also for a better outcome after total knee arthroplasty implantation. Indeed, patellar maltracking is responsible for about half of the poor results in total knee arthroplasty [24].

Several in vitro studies have investigated the influence of the quadriceps muscle on the kinematics and contact forces of the patella. Identifying the QV, however, is very complex, especially in a cadaveric study, and sometimes ambiguous given the lack of standardization of this parameter [12]. For this reason, one or more force vectors representing the action of the quadriceps muscle are often used in in vitro studies. Some studies have divided the quadriceps muscle into different muscle components and applied varying forces to simulate the action of vastus lateralis and medialis and create a more realistic representation of muscle activity [25–29]. However, there is no consensus in the literature regarding the number of sub-components into which the quadriceps should be discretized. This variability introduces inconsistencies and complicates comparisons between studies [30]. Moreover, the increasing complexity of experimental setups, such as defining the direction and force proportion of each muscle component, adds layers of uncertainty and reduces the controllability of the experiments.

Mizuno et al. [10] presented a simplified approach by considering quadriceps force as a single resultant vector, analyzing the impact of different quadriceps directions solely in the frontal plane. While this reduces experimental complexity, it neglects the posterior components of the vastus medialis and lateralis forces, which could significantly influence patellar kinematics and contact forces [26].

Furthermore, to the best of our knowledge, existing studies typically evaluate three main conditions: physiological, lateralized, and medialized QV. These studies introduce alterations within physiological limits to replicate scenarios assessable in clinical practice, therefore neglecting the effects of larger QV deviations. Consequently, it remains unclear to what extent a substantially altered QV leads to pathological kinematics and the nature of the relationship between QV deviations and changes in patellar kinematics.

In summary, while prior studies have advanced the understanding of the relationship between QV and patellar behavior, they show significant limitations. These include the lack of agreement on quadriceps discretization, challenges in experimental controllability due to increased complexity, and restricted scope of QV variations examined. Addressing these gaps could provide a more comprehensive understanding of patellar kinematics in both physiological and pathological conditions.

The purpose of this in vitro study is to fill the gap in the current literature: by systematically varying the quadriceps vector direction in both the frontal and sagittal planes, and spanning a large range of clinically relevant values of quadriceps loads, this research aims to elucidate how the loading configuration influences the behavior of the knee. This approach allows for an exploration of the biomechanical interplay between quadriceps forces and both PF and TF kinematics. This study provides crucial insights for clinicians about the possible consequences of surgical undercorrection, overcorrection, or maltracking.

## Materials and methods

### Specimens

A total of 12 paired human cadaveric lower limbs from six donors were obtained postmortem with the approval of the Bioethical Committee of the University of Bologna (no. 0150450 of 5 June 2023). The donors were all Caucasian, four males and two females (Table 1). Inclusion criteria were: no history of lower limb fractures, no major deformities, donors physically active, engaging in ambulatory activities and daily living tasks up until the date of death. The median age of the subjects at the time of death was 60 years, and their median weight was 108 kg.

**Table 1:** Details of specimens and donors' information

Specimen number	Side	Donor	Sex	Age at death (years)	Height (cm)	Weight (kg)
#1	Left	A	M	43	180	148
#2	Right					
#3	Left	B	F	61	165	98
#4	Right					
#5	Left	C	M	67	188	154
#6	Right					
#7	Right	D	M	51	188	57
#8	Left					
#9	Right	E	M	68	173	118
#10	Left					
#11	Right	F	F	59	170	37
#12	Left					
Median				60	176.5	108
Standard deviation				9.2	45.4	13.7

The specimens were acquired through an ethically approved international donation program (Anatomy Gift Registry, Hanover, MD, USA). To preserve their mechanical properties, the specimens were kept hydrated during the tests and then stored at −28 °C. They were thawed at room temperature 24 h before the trials and tested at room temperature with water vaporization during the tests.

### Specimen preparation and imaging

The skin, underlying fat, and muscles other than the distal quadriceps were removed by orthopedic surgeons, with care taken to preserve the joint capsule, the retinaculum, and the quadriceps muscle, fascia, and tendon. The quadriceps tendon was left intact and sutured to provide firm attachments for the clamping system to connect the loading cables.

To grant reproducible test conditions, the anatomical axes of the specimens were measured according to the procedure described in reference [31]. The proximal and distal ends of each limb were cut and embedded with acrylic cement. Resections were performed at 60% of the diaphysis of the femur and tibia, starting from the distal and proximal sides, respectively. The cement pots, which were cylindrical in shape, were positioned so that their axis corresponded with the longitudinal anatomical axes of the tibia and femur. To preserve the mutual distance between the tibia and fibula, the specimens were cut and embedded while still frozen.

A total of three stainless steel screws were placed, not aligned and not equidistant with each other, on the femur, tibia, and patella. These screws served as reference points for subsequent data analysis. Owing to the small geometry of the patella, the three screws also served to house a holder for the patellar tracker (see below). Each specimen was scanned with computed tomography (CT; GE Discovery CT scanner, GE Healthcare, Milwaukee, WI, USA; slice thickness = 0.625 mm, in-plane resolution = 0.781 mm) twice: when still intact and after the preparation described above (i.e., tissue removal, bone cutting, cement embedding, and screw positioning).

### Loading rig

**Fig. 1:**
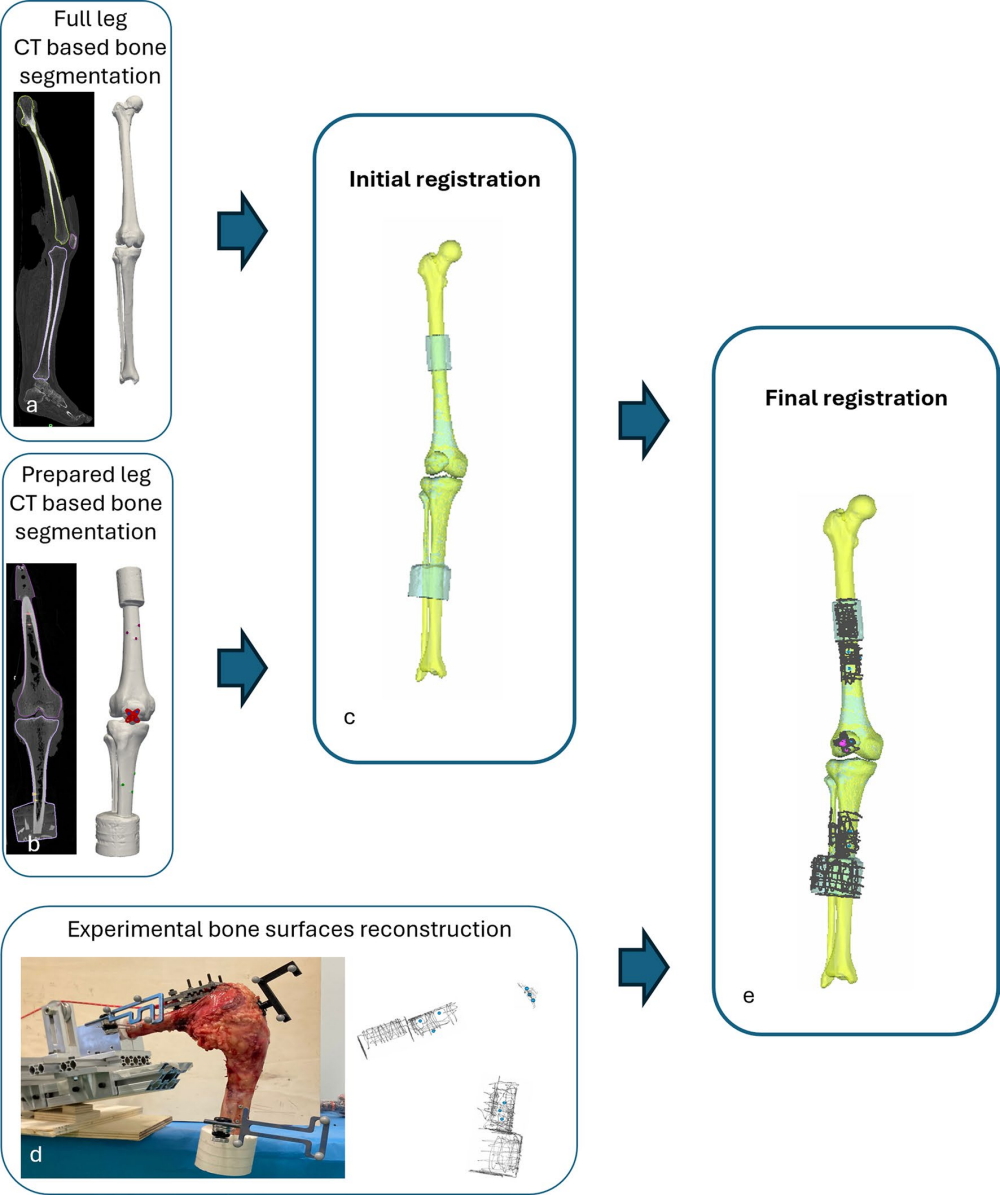
Overview of the registration procedure applied to the images and to the experimental testing. From the CT scan of the intact leg, the 3D bone surfaces were obtained through segmentation (**a**). A second CT scan was performed after specimen preparation to acquire the bone surfaces as used in the experimental setup (**b**). The two segmentations were initially registered to align the bone surfaces (**c**). During the experimental phase, bone surfaces of the tibia and femur, as well as a dedicated patellar holder, were reconstructed in the experimental reference frame using a specialized pointer (**d**). These reconstructions were then registered with the segmented bone models (**e**) to determine the transformation from the experimental to the anatomical reference frame. Figshare repository (https://doi.org/10.6084/m9.figshare.29278715)

A testing rig was constructed to hold the specimens during measurements of PF and TF kinematics while flexing the knee under different loads applied to the quadriceps. The femur was fixed while the tibia hung freely (Fig. 1d). The equipment allowed adjustment of all degrees of rotation and translation of the femur, allowing fixation in accordance with the subject’s specific alignment. To minimize weight-induced torque, the internal–external rotation of the femur was chosen so that the tibia hung vertical, under only the effect of gravity, thus avoiding any kinematic forcing. The knee joint was also centered with respect to the focus of the optoelectronic system cameras (see below). The rig was designed to maximize the visibility for the optoelectronic tracking system, minimizing marker occlusion artifact [32]. The knee was positioned with the patella in the superior position to prevent gravity from affecting its kinematics. A clamping system was attached to the sutured tendon to deliver the force. To ensure that a constant force was applied, static weights were connected to the clamp by ropes. The muscle direction was adjusted by a system of adjustable pulleys on the rig frame. The quadriceps tension pulled the knee toward the extended position. The tibia flexion was controlled by the operator with a pushing rod in a single point of contact with the cylindrical cement pot, thus introducing a single force which constrained only the flexion, while the remaining motion components were determined by the equilibrium of the joint constraints under the applied quadriceps loads.

### Motion acquisition

Trackers with triplets of passive markers were attached to the femoral diaphysis, to the anterior side of the patella (using the previously mounted support), and to the proximal metaphyseal–diaphyseal junction of the tibia. A pointer, also embedding three passive markers, was used to digitally reconstruct the bone surface and the centers of the previously placed screws as clouds of points (Fig. 1d). Marker movement was measured using an optoelectronic system (Vicon Motion Systems Ltd.; nominal accuracy 0.5 mm/0.5°). The system was composed of eight cameras, of which six were VICON Bonita 10 (1024 × 1024-pixel, 250 Hz) and two VICON Vero (2048 × 1024-pixel, 330 Hz). The arrangement of the cameras was designed to maximize system accuracy [32].

### Experimental method

**Fig. 2:**
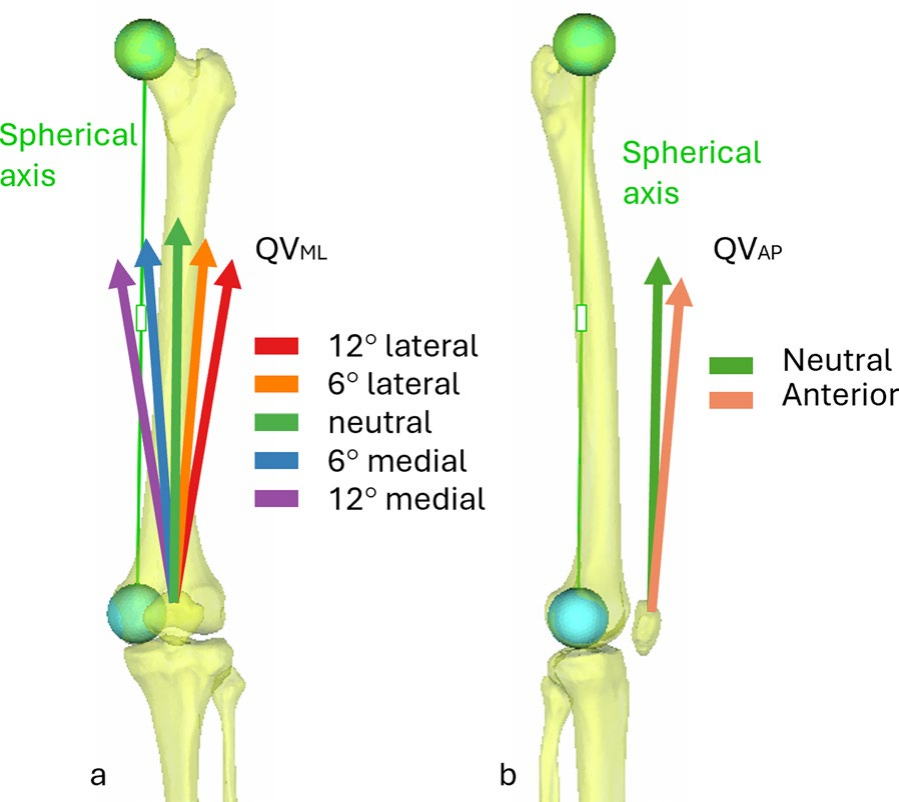
Variations of quadriceps direction in the frontal (**a**) and sagittal (**b**) plane. Figshare repository (https://doi.org/10.6084/m9.figshare.29278715)

To simulate different activation patterns of the quadriceps muscle, three parameters were varied: the force magnitude of the quadriceps vector (QV_load_), the medial–lateral direction of the quadriceps load line in the femur frontal plane (QV_ML_), and the anterior–posterior direction of the quadriceps load in the femur sagittal plane (QV_AP_). Three different QV_load_ were used: 20 N (a minimum preload that kept the patella in its physiological pose), and 160 N and 280 N (to simulate two levels of muscle contraction). The direction of the quadriceps action line was adjusted from a neutral condition. The neutral condition was defined so that the QV line was parallel to a line from the center of the spherical femoral head to the center of a sphere fitted on the medial condyle. This line is often referred to as the spherical axis [33]. Five QV_ML_ conditions were simulated (Fig. 2a) by medializing and lateralizing the load line by 6° and 12° to cover both the physiological and the pathological range. Two QV_AP_ conditions were simulated (Fig. 2b), 0° and 5° anterior, from the neutral direction. In total, ten loading directions were thus tested on each specimen, each at three force levels, resulting in a total of 30 tests per specimen.

For each test, the starting position was defined as the maximum flexion reached when the operator pushed on the tibia. The ending point was defined as the maximum extension reached under the effect of the quadriceps load. The range therefore varied between specimens, depending on the test. Each knee was then cyclically extended and flexed three times, followed by a recovery period of approximately 1 min. The optoelectronic system tracked the position of the femur, tibia, and patella throughout the test.

### Data analysis

Knee joint motion was analyzed with the proprietary software of the optoelectronic system (Nexus 2.5, Vicon), which allowed for the reconstruction of marker trajectories throughout the test sequences. To evaluate the relative motion of bone segments, the data obtained were transformed from the experimental reference frame to anatomical reference frames (Fig. 1). The full transformation process is described in detail in Supplementary Material 1; a summary is provided here.

From the CT scans acquired before and after surgical preparation, the bone surfaces (femur, tibia, and patella) were obtained using a semi-automatic segmentation tool (Mimics, vers. 25.0, Materialise NV). Anatomical reference frames were defined on the segmentations obtained from the intact leg on the basis of identifiable landmarks and following established conventions [34].

To allow kinematic tracking after surgical preparation, the segmented models from pre- and postoperative scans were aligned using an automated registration procedure (Fig. 1c). The position of the anatomical frames was then mapped onto the experimental reference system through an additional registration step, which matched the segmented geometries with the digitized point clouds obtained from the motion capture system (Fig. 1d).

**Fig. 3:**
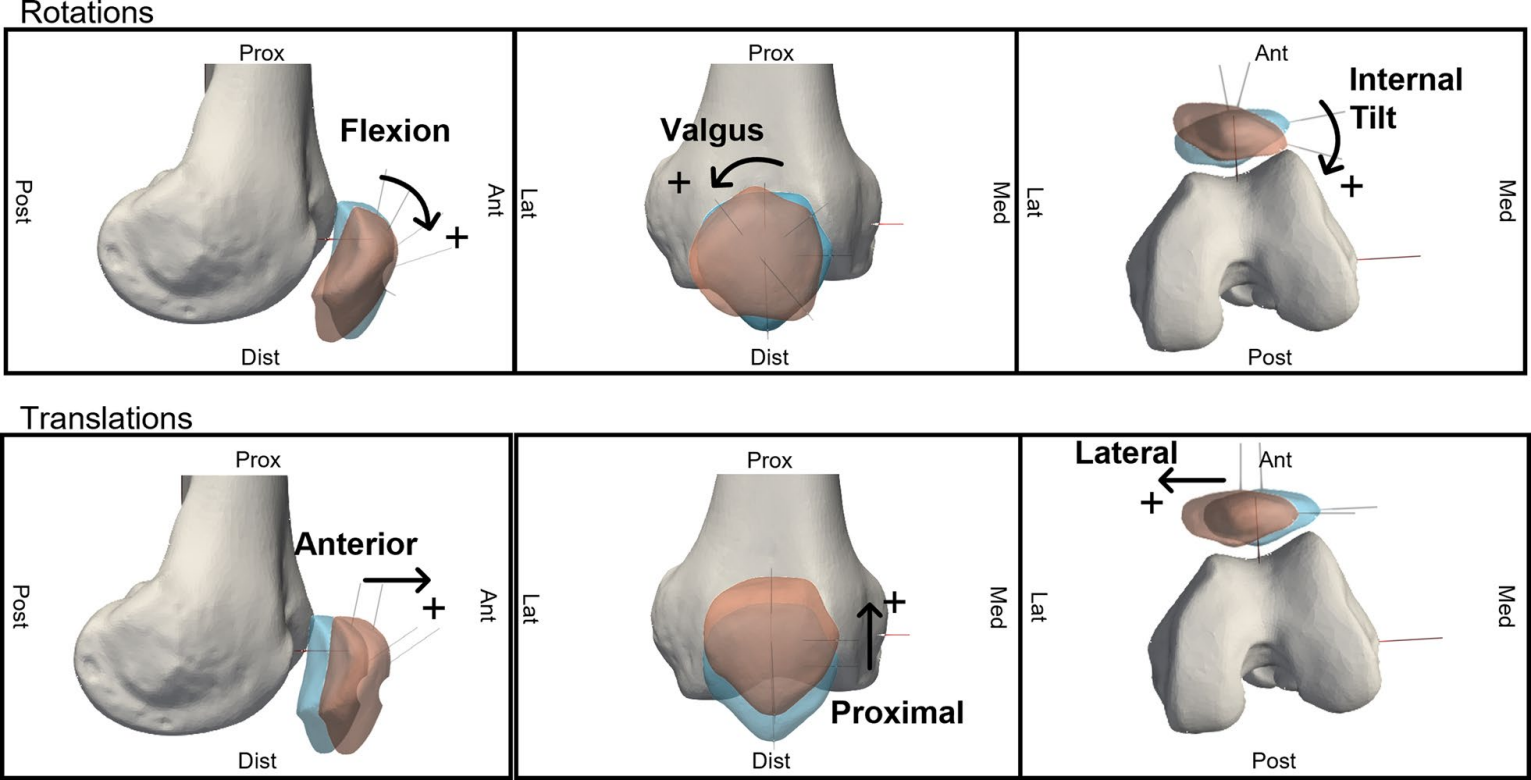
Motion components of the patellofemoral joint of a right leg. Figshare repository (https://doi.org/10.6084/m9.figshare.29278718)

This process enabled the definition of transformation matrices, which were used in MATLAB (vers. 2023b, MathWorks Inc.) to calculate joint kinematics. The relative motion of the patella with respect to the femur and of the femur with respect to the tibia were quantified, using the Grood and Suntay convention [35] to describe motion in terms of three rotations and three translations (PF joint in Fig. 3, TF joint in Supplementary Figure S4_1).

For each specimen, the condition with neutral quadriceps force direction and a 20-N load was used as a reference. Comparisons with conditions involving different force directions and magnitudes allowed for assessment of their specific impact on joint kinematics, independent of intersubject variability. The effect of these variations was quantified by analyzing the differences in joint motion relative to the reference curves.

### Statistical analysis

To ensure that the baseline condition (reference test) did not contain outliers that could bias the analysis, Pierce’s test was applied to the kinematic variables across all specimens. No outliers were identified.

The Shapiro–Wilk test indicated that the data did not follow a normal distribution. To analyze the statistical significance of the effect of QV_load_, QV_ML_, and QV_AP_, the Kruskal–Wallis test was applied to the variation from the reference test of each component of motion of both joints throughout knee flexion at 1° intervals. Given the large number of repeated comparisons, Bonferroni correction was applied to adjust for multiple testing; the significance threshold was set at *α* = 0.05, and the corrected *p*-value threshold was obtained by dividing *α* by the number of comparisons.

In addition, to explore potential interaction effects between load magnitude and vector direction, a secondary analysis using linear mixed-effect models was performed. Since this type of analysis is less robust than the Kruskal–Wallis statistical analysis, the results relating to the mixed-effect model are reported in the Discussion section as complementary to the main analysis.

All statistical analyses were performed using R (R version 4.0.1, http://www.r-project.org).

## Results

Measurements were successfully obtained for all loading conditions and specimens, except for specimens #11 and #12 from the same donor. For these specimens, the tests at 12° medial could not be performed to avoid damaging the capsule, as the patella tended to dislocate.

As explained above, the range from maximum extension to maximum flexion of the tibia varied between specimens and depending on the test. For the neutral condition and 20 N, the maximum tibiofemoral extension was 1.2° ± 7.5° (median and standard deviation between all specimens), while the maximum flexion was 125.5° ± 12°. Higher loads reduced maximum flexion to 119.8° ± 10.3°.

First, the kinematics for the reference condition (which corresponded to neutral QV angles and 20 N force) were computed. They are reported in detail in Supplementary Material 2.

### Patellofemoral joint

To evaluate the effect of different parameters applied to the quadriceps muscle, as previously noted, mean values, standard deviations, and statistical tests were calculated across all specimens and categorized by each QV parameter (QV_ML_, QV_AP_, QV_load_), considering the variations with respect to the neutral kinematics.

**Fig. 4:**
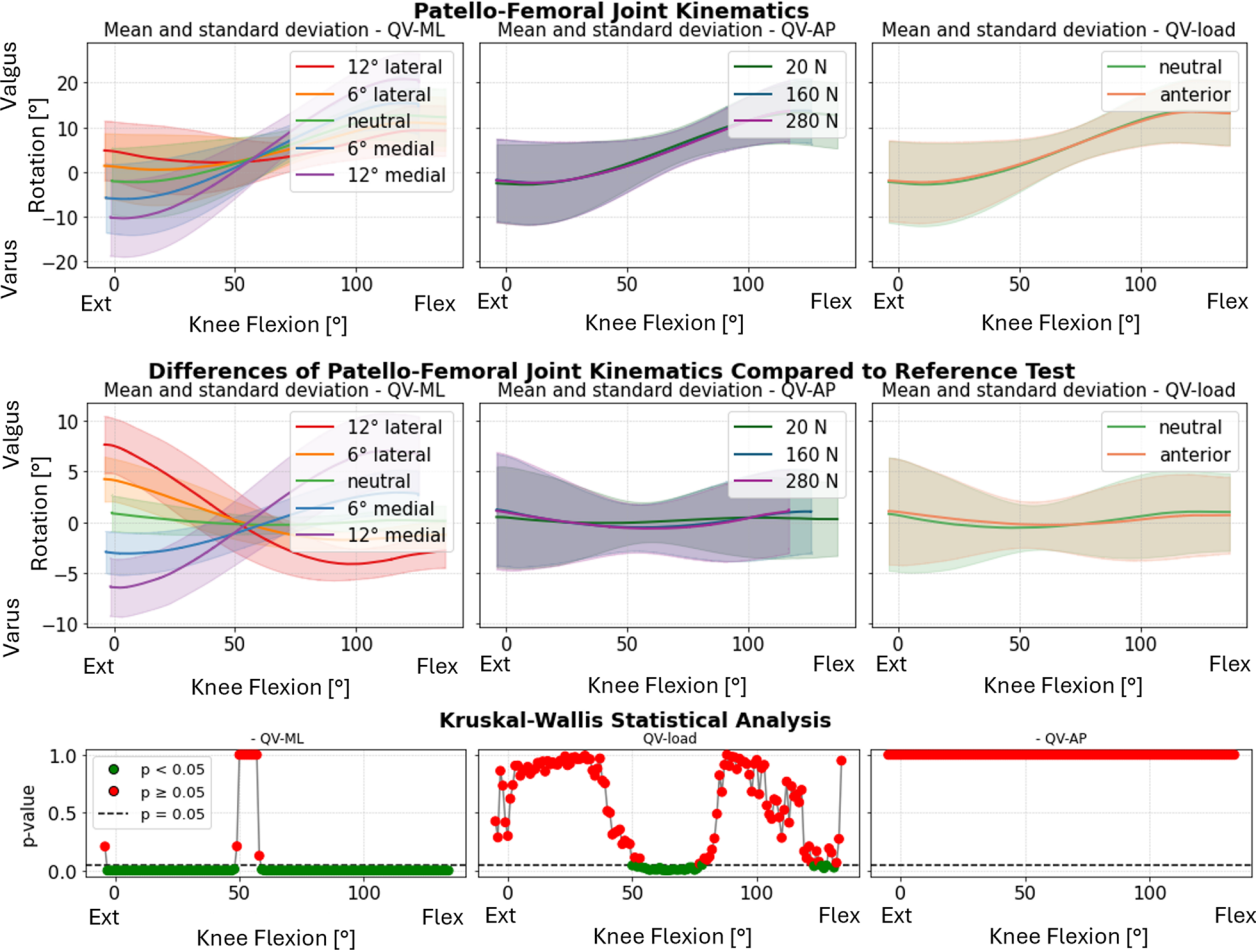
Varus–valgus rotation of the patella. Top: absolute values as a function of knee flexion angle (the median and standard deviation between 12 specimens are plotted). Center: differences of all tests compared with the reference test (QV_load_ = 20 N, QV_ML_ = neutral, QV_AP_ = posterior). Left shows the difference as a function of QV_ML_, middle QV_load_, and right QV_AP_. Bottom: significance of the differences plotted at the center. The *p*-value trend is plotted for the three parameters (left QV_ML_, middle QV_load_, and right QV_AP_); the significant values are highlighted in green (*p* < 0.05) and the nonsignificant ones in red (*p* ≥ 0.05)

Statistical analysis indicated that the QV_ML_ had the most significant impact on PF kinematics. This influence was particularly pronounced in varus–valgus rotation (Fig. 4, first column), where lateralizing the load induced varus rotation in full extension, while medializing the load resulted in valgus rotation. Interestingly, this behavior reversed at full flexion, with lateralization leading to valgus and medialization causing varus. In all the specimens, the inversion happened within a small flexion range, around 55°: for that angle, the patellar motion became independent of the QV_ML_.

In contrast, the QV_load_ (Fig. 4, second column) exhibited a less evident effect, noticeable only around mid-flexion angles (approximately 50–80°). The anterior–posterior direction (QV_AP_) (Fig. 4, third column) did not show a statistically significant influence on patellar abduction–adduction angles.

**Fig. 5:**
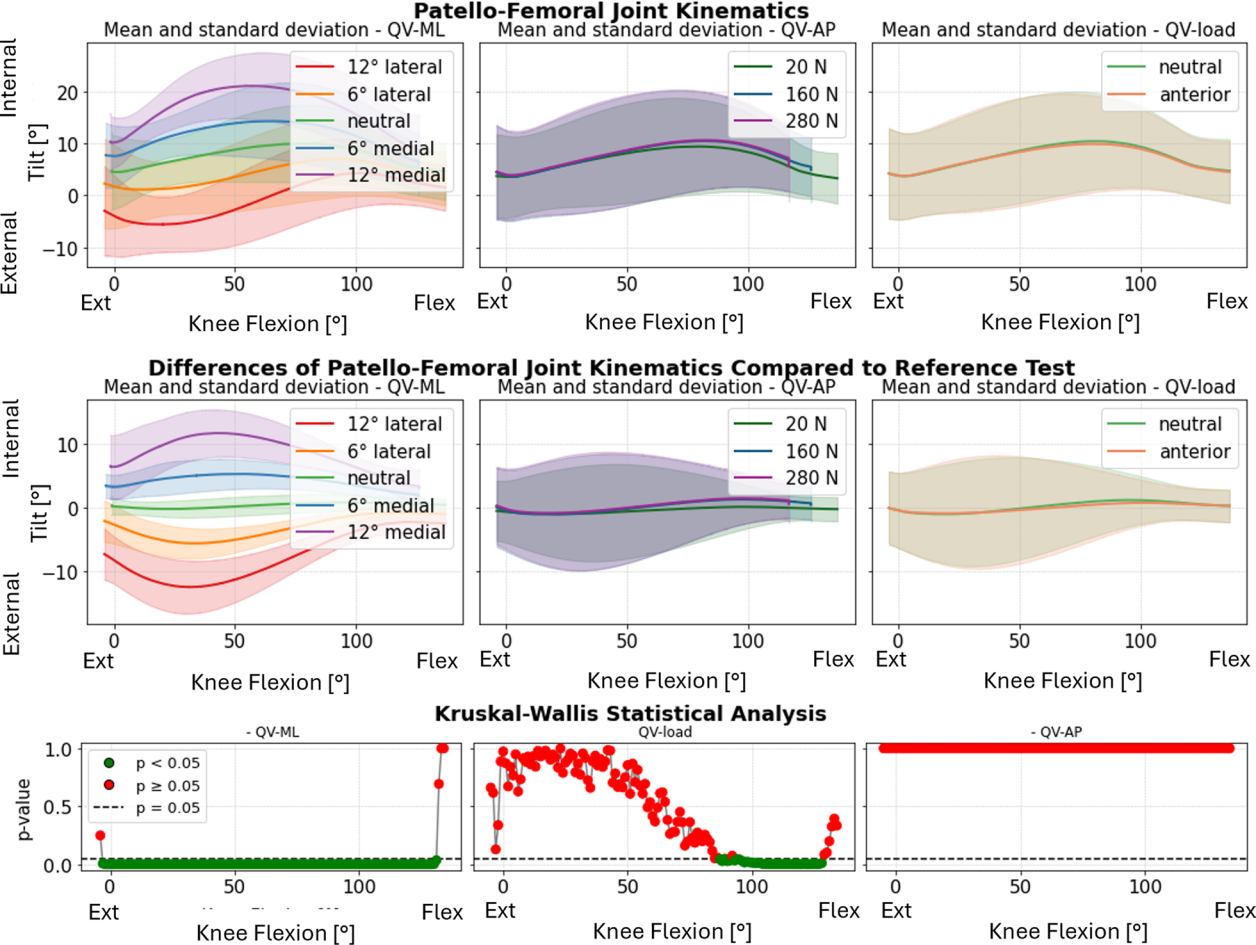
Internal–external tilt of the patella. Top: absolute values as a function of knee flexion angle (the median and standard deviation between 12 specimens are plotted). Center: differences of all tests compared with the reference test (QV_load_ = 20 N, QV_ML_ = neutral, and QV_AP_ = posterior). Left shows the difference as a function of QV_ML_, middle QV_load_, and right QV_AP_. Bottom: significance of the differences plotted at the center. The *p*-value trend is plotted for the three parameters (left QV_ML_, middle QV_load_, and right QV_AP_); the significant values are highlighted in green (*p* < 0.05) and the nonsignificant ones in red (*p* ≥ 0.05)

The patellar tilt also showed significant sensitivity to QV_ML_, while QV_AP_ and QV_load_ had minimal effects (Fig. 5). At full flexion, the differences between medial and lateral directions were less pronounced but remained distinct. Medial QV_ML_ induced internal tilt throughout the flexion range, whereas lateral QV_ML_ caused external tilt. The most prominent differences were observed between 20° and 60° of knee flexion.

**Fig. 6:**
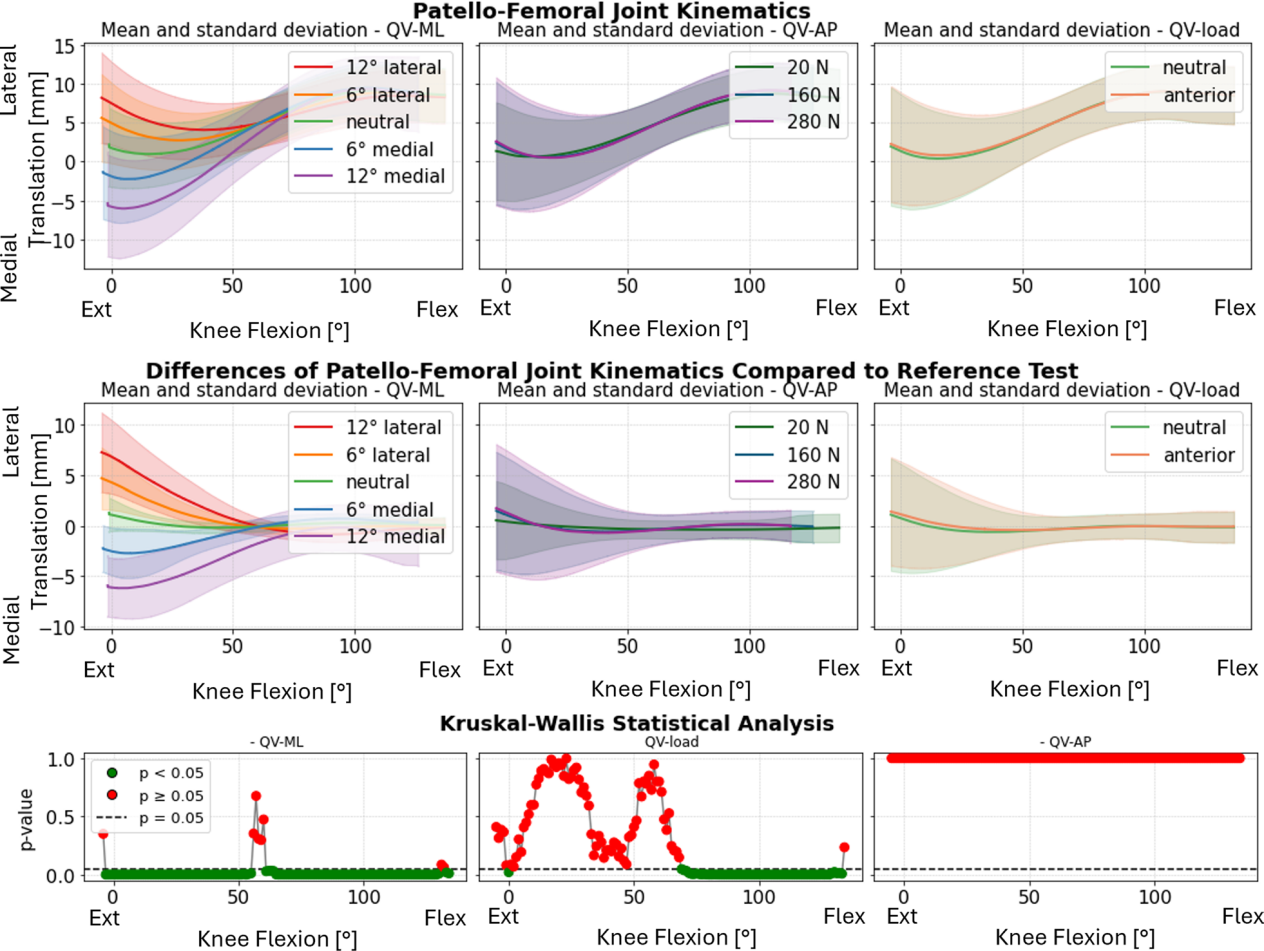
Medial–lateral translation of the patella. Top: absolute values as a function of knee flexion angle (the median and standard deviation between 12 specimens are plotted). Center: differences of all tests compared with the reference test (QV_load_ = 20 N, QV_ML_ = neutral, and QV_AP_ = posterior). Left shows the difference as a function of QV_ML_, middle QV_load_, and right QV_AP_. Bottom: significance of the differences plotted at the center. The *p*-value trend is plotted for the three parameters (left QV_ML_, middle QV_load_, and right QV_AP_); the significant values are highlighted in green (*p* < 0.05) and the nonsignificant ones in red (*p* ≥ 0.05)

Medial–lateral translation was significantly affected by QV_ML_, particularly at full extension. The differences became smaller after 60° knee flexion. QV_AP_ and QV_load_ showed minimal influence on this parameter (Fig. 6). For one specimen (specimen #6) the patella did not correctly engage in the trochlea when QV_ML_ 12° medial was applied.

Flexion–extension, anterior translation, and proximal translation of the patella were mainly influenced by QV_AP_ and the QV_load_, but only at full extension. Their trend is reported in the Supplementary Material (Supplementary Figure S3_8–13). In Supplementary Material 3, the absolute PF kinematics (without subtracting the reference test in neutral condition) of specimen #1 is reported for all tests performed, grouped by parameter (Supplementary Figure S3_2–7).

### Tibiofemoral joint

**Fig. 7:**
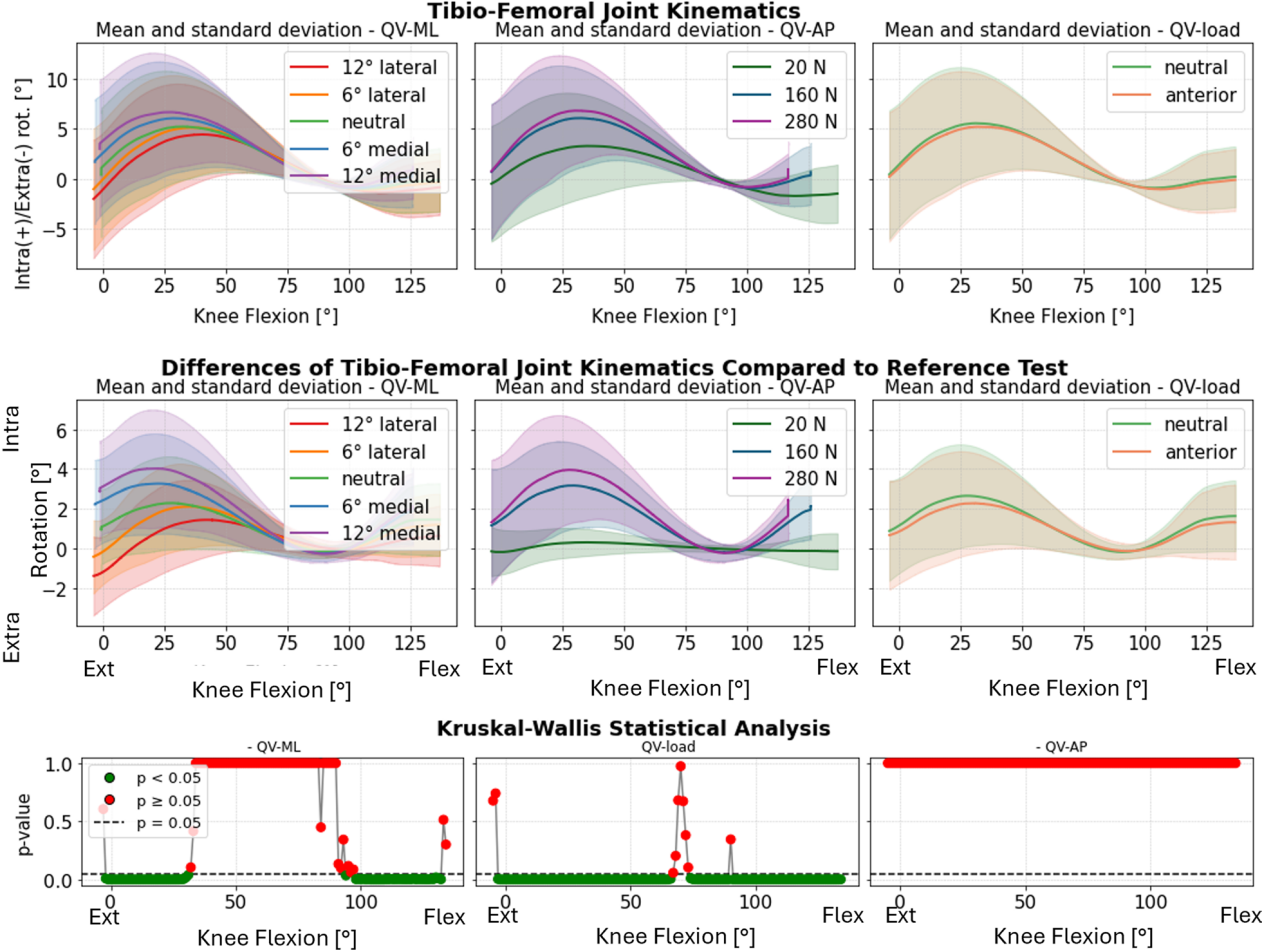
Intra–extra rotation of the femur. Top: absolute values as a function of knee flexion angle (the median and standard deviation between 12 specimens are plotted). Center: differences of all tests compared with the reference test (QV_load_ = 20 N, QV_ML_ = neutral, and QV_AP_ = posterior). Left shows the difference as a function of QV_ML_, middle QV_load_, and right QV_AP_. Bottom: significance of the differences plotted at the center. The *p*-value trend is plotted for the three parameters (left QV_ML_, middle QV_load_, and right QV_AP_); the significant values are highlighted in green (*p* < 0.05) and the nonsignificant ones in red (*p* ≥ 0.05)

The TF joint was significantly affected in all motion components by QV_load_. In particular, the greatest differences are seen between passive flexion (20 N) and other loads, but not particularly between 160 N and 280 N. The two rotations (abduction–adduction and internal–external rotation), especially with the leg extended, were also affected by QV_ML_, but in a more attenuated manner than the PF joint. QV_AP_ showed no significance in any motion components (Fig. 7).

Plots representing the mean and standard deviation of the kinematic differences of the TF joint are shown in Supplementary Material 4 (Supplementary Figure S4_7–11). For each motion component, the results are grouped considering the three QV parameters (QV_ML_, QV_load_, and QV_AP_). In addition, for each of these plots, the *p*-value trend is shown, indicating the significance of that parameter in influencing TF kinematics. In Supplementary Material 4, the absolute TF kinematics (without subtracting the reference test in neutral condition) of one specimen is reported for all tests performed, grouped by parameter (Supplementary Figure S4_2–6).

## Discussion

The primary goal of this study was to provide a more comprehensive understanding of how alterations in the direction and magnitude of the quadriceps force affect PF and TF joint biomechanics. These findings offer valuable insights for clinical and surgical management of patellar disorders [36] and may help minimize complications from mispositioned knee implants [37]. To this aim, in vitro tests were conducted using 12 lower limbs from human cadavers. Quadriceps force variations were systematically applied across three parameters of the quadriceps vector: mediolateral (QV_ML_) and anteroposterior (QV_AP_) directions as well as load magnitude (QV_load_). These tests simulated both physiological and pathological conditions over the full range of knee flexion. Bone motion was tracked using stereophotogrammetry.

In the reference conditions (QV_load_ = 20 N, QV_ML_ = 0°, and QV_AP_ = 0°), the observed flexion range (125.5° ± 12°) was similar to the physiological ranges reported in the literature [38]. Increasing the quadriceps force reduced the maximum flexion angles. This is consistent with the biomechanical principle that increased forces restrict joint mobility, likely owing to the increased tension in surrounding soft tissues [39].

Comparison with the literature indicates that the kinematic results in reference conditions aligns well with previously reported ranges of kinematics. Both PF [25] and TF [40] kinematics in reference conditions showed trends and ranges of values similar to those shown in the literature. Furthermore, the low interspecimen variability confirmed the reproducibility of the assessment of the patellar motion.

### Patellofemoral joint

The tests in the reference condition showed that the PF joint translations are highly repeatable during movement, in fact, the standard deviation was low throughout the movement (Supplementary Material 2, Supplementary Figure S2_1). Only one specimen showed a different behavior in the medial–lateral translation, showing an almost null variation from the extended to the flexed knee. A retrospective analysis of the CT scans of this specimen revealed a case of patella baja. This behavior could be thus attributed to the fact that, given this condition, the patella is engaged in the trochlea at lower levels of flexion than normal, and therefore the constraint imposed by the trochlea already acts at nearly extended leg. This increased constraint in the patella baja condition is also reflected in the varus–valgus curves. In any case, the effects on the kinematics of the patella baja condition are not the subject of this study, and we do not have sufficient numerosity to draw any conclusions. When evaluated in the baseline condition, the samples were not considered as outliers following the Pierce test.

Regarding the PF joint rotations, a greater variability in the magnitudes of the angles was visible, while preserving a very similar trend of the curves in all the specimens. The range measured in this study was similar to those previously reported in literature [25].

One of the crucial results of this investigation is the quantification of how QV variations affect the medial–lateral translation of the patella (Fig. 6). For all QV_ML_ conditions, the differences in medial–lateral translation were maximal at full extension and diminished up to 50–60° of flexion. The increased translation in early flexion indicated that the patella was more susceptible to medial–lateral displacement at this stage when it was not fully engaged in the femoral trochlea yet [41]. Beyond 60°, the joint stabilized, probably owing to a greater geometric constraint. In this condition, it is very likely that QV_ML_ variations would result in different intensity and distribution of contact pressure. Future investigations will aim to further investigate this aspect by introducing an instrumented patellar prosthetic component capable of measuring medial and lateral reaction forces at the PF joint using force sensors [42].

QV_ML_ highly affected patellar rotations, especially varus–valgus rotation and internal–external tilt. In particular, the mean curves showed marked differences at 12° (medial and lateral) compared with neutral (Fig. 5). Smaller QV_ML_ angles (6°, both medial and lateral) resulted in less marked effects on the patellar rotations. This suggests that smaller changes in QV direction generate little kinematic perturbation compared with the reference condition, but still significant. Furthermore, the symmetrical behavior between medial and lateral directions suggests that quadriceps displacement acts directly and proportionally on the kinematics of the patella.

**Fig. 8:**
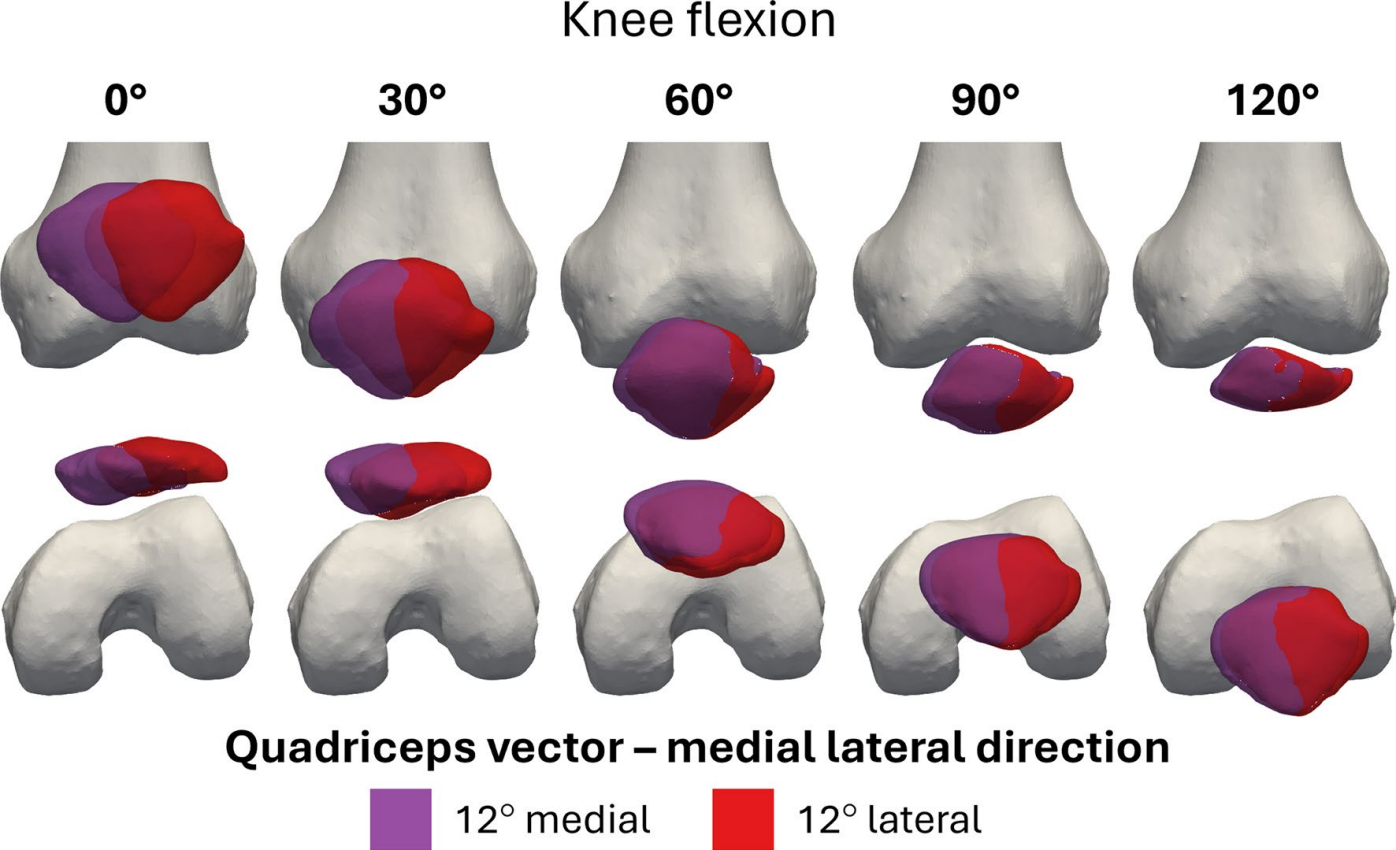
Reconstruction of the patellar motion on the femur at 30° intervals of knee flexion for the test conducted with QV_load_ = 20N, QV_AP_ = 0°, and QV_ML_ = 12° medial in purple, 12° lateral in red

The difference due to QV_ML_ in varus–valgus rotation (Fig. 4) was significant throughout the range of knee flexion, except for angles between 50° and 60°, where an inversion of the kinematic trend occurred. Medial QV deviations caused valgus rotation at full extension, transitioning to varus rotation beyond 60° of flexion, with the opposite pattern for lateral QV deviations. Around 50–60° flexion, the patella stabilized within the trochlear groove, suggesting a peak in joint congruence where the surfaces match optimally, constraining relative motion. This flexion range appears to represent a mechanical equilibrium where the QV, deviating medially or laterally, interacts more directly with the trochlear walls [3], reversing the trajectory. The fact that the opposite occurred in such a defined range of flexion, as well as the convergence in medial–lateral translation, suggests a highly constrained biomechanical behavior and is probably related to common anatomical features among the specimens. This effect was also visible when the kinematics were analyzed through the helical axis (see Supplementary Material 5). In particular, it was noted that the helical axes diverged for low degrees of flexion, particularly in the pathological condition of QV_ML_. From 60° onward, however, the helical axes of all directions converged (Fig. 8).

QV_ML_ had a predictable influence on the internal–external tilt of the patella (Fig. 5), with lateral displacements promoting more external tilt and medial displacements promoting more internal tilt with respect to the neutral direction. This behavior may be clinically relevant, as significant alterations in patellar tilt are associated with pathological conditions such as PF pain syndrome and current lateral patellar dislocation [43]. The clinical literature indicates that lateral dislocation is more common compared with medial dislocation [44]. In our study, strong instabilities on the medial side were more common, but they did not lead to dislocation. In specimen 6, a 12° medialization of QV caused severe patellar maltracking. The patella did not dislocate, but was not correctly engaged in the trochlea, even at higher degrees of flexion. Medial instability was also noted with specimens 11 and 12 already at 6° medial QV_ML_. These specimens were affected by the condition of patella baja. The instability was stronger under higher QV loads, even though QV_load_ alone had limited statistical significance for medial–lateral translation. QV_load_ consistently amplified the effects of QV_ML_ deviations. Although the effects of QV_ML_ and QV_load_ were analyzed independently, some combinations—such as high QV_load_ under medialized directions—appeared to amplify patellar maltracking more than either factor alone. This suggests the presence of possible interaction effects between vector direction and load.

A secondary analysis using mixed-effect models was conducted to explore potential interactions between QV direction and load magnitude on patellar kinematics. The interaction effects were most pronounced in the internal–external rotation during mid-flexion angles. Specifically, as the load increases, the significance of the interaction between load and QV direction at 12° medial and lateral deviation also increases. For internal–external rotation, significant interactions (< 0.001) were observed at medial 12° QV deviations between 25° and 40° flexion at 160 N load, and from 15° to 50° flexion at 280 N load. At lateral 12° deviation, significant interactions occurred from 30° to 50° flexion at 160 N and from 20° to 50° and around 90°–100° flexion at 280 N load. Regarding medial–lateral translation, strong interaction effects were found between load and QV direction for both 12° medial and lateral deviations. These interactions were significant from 0° to 70° flexion at 160 N load and extended from 0° to 90° flexion at 280 N load. Overall, patellar kinematics during mid-flexion appear strongly modulated by the interplay between load and quadriceps vector direction in extremely medialized or lateralized quadriceps directions, while no significance was found for the other conditions.

### Tibiofemoral joint

For the TF joint, QV_load_ was the most influential parameter, with statistically significant differences across all motion components, especially between passive flexion (20 N) and active contraction (160 N and 280 N). Both QV_AP_ and QV_ML_ had no significant effects on TF translations. QV_ML_ significantly influenced the rotations, but to a lesser extent than for the PF joint. This suggests a relevant biomechanical interaction between the two joints during knee flexion. The tibia and the patella are loaded in series by the quadriceps. However, as part of the force is transferred from the patella to the femur, only part of the forces generated by the quadriceps vector are actually transferred to the tibia, thus reducing the direct impact on the tibia [25, 45, 46]. At the same time, this coupling suggests that PF-joint malalignment could also indirectly influence TF-joint mechanics, emphasizing the need for holistic assessments and treatments targeting both joints, so as to avoid pathological loading [47].

### Limitations

This study involved 12 paired specimens, which may represent a relatively small sample size. However, this limitation is inherent to cadaveric studies due to the high costs, logistical complexity, and ethical considerations associated with specimen preparation and testing. While the relatively small sample size may limit the statistical power for detecting more subtle differences, the use of intraspecimen comparisons helps reduce variability and strengthen the interpretability of the observed effects. Furthermore, the inclusion of paired specimens from the same donor may have influenced the results.

A key limitation is the application of quadriceps forces that are lower than those experienced during in vivo daily or athletic activities, where quadriceps loads can exceed several times body weight. However, this choice was necessary to prevent damage to the specimens—particularly at the patellar tendon interface or within the clamping systems—which is a common constraint in cadaveric experimental setups. Nonetheless, in this study, loads up to 280 N were applied, exceeding the commonly used value of 175 N [25, 26, 28, 29, 48–50]. Moreover, three distinct load levels were analyzed to investigate potential load-dependent trends in kinematic behavior. Three load value trends were analyzed with the implicit assumption of capturing the non-linearity of the response at low loads, while also assuming that increasing the load results in greater linearity. Absolute values of joint motion may differ under higher physiological loads. Although observed patterns and directional effects may provide relevant biomechanical insights, subphysiological loads affect the generalizability of these findings to clinical practice or in vivo conditions.

Another potential limitation is the simplification of the quadriceps muscle as a single line of action. This approach, already widely used in the literature [10, 26, 27, 51–55], was adopted to reduce experimental uncertainties and increase control over the applied parameters. The representation of the quadriceps as a single force captures the natural convergence of quadriceps forces through the patella, reflecting its mechanical role as a pulley [56] and being dynamically equivalent to the application of separate quadriceps forces. However, it should be acknowledged that this approach makes less evident the differentiation of the specific mechanical roles of the individual quadriceps components—particularly the vastus medialis and lateralis—which are known to play distinct roles in mediolateral patellar stability. While some studies [10, 25–27] used different numbers of forces to model quadriceps action, our simplified setup allowed a more controlled analysis of how quadriceps direction affects knee motion.

Despite these simplifications, the results show strong agreement with previously published literature. Shalhoub et al. [26] compared single- and multi-vector approaches and suggested that posterior quadriceps components have limited influence on deep flexion kinematics, which are mainly governed by joint anatomy. Similarly, we found that QV_AP_ variations had a limited impact on PF kinematics and only at the extended knee.

## Conclusions

The purpose of this study was to evaluate the impact of quadriceps action on the kinematics of the patellofemoral and tibiofemoral joints. A wide range of quadriceps vector directions were analyzed in both the frontal and sagittal planes. The results demonstrate that the kinematics of the patellofemoral joint are highly sensitive to variations in the medial–lateral orientation of the quadriceps vector, while alterations in the anteroposterior direction and the magnitude of the applied load have a more limited effect. The relation between QV orientation and patellar tracking highlights the clinical relevance of accurately assessing quadriceps force direction. Even small angular changes (e.g., ±6°) can substantially alter patellar tracking and tilt, emphasizing the clinical importance of accurately assessing and adjusting quadriceps alignment to prevent complications such as increased contact forces or abnormal wear. Additionally, the role of the patella as a key mediator in force transmission underscores its importance in maintaining overall knee stability and preventing pathological loading of the tibiofemoral joint. Finally, the dependency of the tibiofemoral kinematics on the magnitude of the applied load, more than the direction of the load, highlights the distinct biomechanical behaviors of these joints. This study contributes to a deeper understanding of the relationship between the QV and knee joint biomechanics, with potential applications for both PF pain management and surgical planning. These findings address key gaps in the literature by analyzing quadriceps force modifications not only within physiological limits but also beyond, providing insights into extreme conditions that could lead to complications such as excessive medialization. Specifically, results suggest that carefully calibrated adjustments to the quadriceps alignment could mitigate patellofemoral malalignment while preserving tibiofemoral joint stability. In conclusion, this study provides a comprehensive framework for understanding the interplay between quadriceps force direction and knee joint biomechanics, paving the way for more targeted interventions in the treatment of patellofemoral joint disorders in the native knee and in total-knee-replacement cases.

## Supplementary Information

Supplementary Material 1.

## Data Availability

The data that support the findings of this study are available upon reasonable request.

## Abbreviations

PF: Patellofemoral
TF: Tibiofemoral
QV: Quadriceps vector
QV_ML_: Medial–lateral direction of the quadriceps vector
QV_AP_: Anterior–posterior direction of the quadriceps vector
QV_load_: Force magnitude of the quadriceps vector
