# Supplementary material for: Quadriceps force direction affects patellofemoral kinematics without impacting tibiofemoral stability: a cadaveric study
Source: Knee Surg Relat Res. 2025 Aug 28;37:36. doi: 10.1186/s43019-025-00286-1 (PMC12395792; doi:10.1186/s43019-025-00286-1)
Supplement: Supplementary file 1 — Supplementary Material 1. [file 43019_2025_286_MOESM1_ESM.docx]

# SUPPLEMENTARY MATERIALS

Appendix to the paper:

Quadriceps force direction affects patellofemoral kinematics without impacting tibiofemoral stability: a cadaveric study

## **SUPPLEMENTARY MATERIAL #1**

### **Details of data analysis**

CT scans with intact leg and after preparation (resection of extremities, embedding in cement, screws positioning) were obtained. From these we obtained the bone surface of the intact femur, tibia and patella and the surfaces of the bone segments used in the experimental phase, as well as the geometry of the cement pots and the position of the screws. In addition, for the patella, the geometry of the marker holder placed during preparation was recreated.

To obtain the bone surfaces before and after preparation, the CT scans were segmented using a semi-automated tool (Mimics 25.0, Materialise, Leuven, Belgium).

From the CT segmentation performed prior to preparation, with the intact leg, anatomical landmarks were identified for the reconstruction of the reference systems (FigureS1_1) according to Anderst et al. [1]. Specifically, the center of the femoral coordinate system was positioned at the midpoint between the epicondyles, with the x- axis perpendicular to the plane on which the two epicondyles and the femoral head lie, with a positive anterior direction. The y-axis connected the center of the reference system with the center of the femoral head, and the z-axis was perpendicular to the other two, with a positive right direction.

For the tibial reference system, the center was placed at the deepest point of the intercondylar eminence. The x-axis was perpendicular to the plane passing through the most medial and the most lateral points of the tibial plateau and the midpoint of the malleoli, with a positive anterior direction. The y-axis connected the midpoint between the malleoli with the midpoint between the two condyles, with a positive proximal direction. The z-axis was perpendicular to the other two, with a positive direction to the right.

For the patella, the origin of the reference system was placed at the midpoint between the most lateral and medial points. The x-axis was perpendicular to the plane passing through the most distal, medial, and lateral points, with a positive anterior direction. The y-axis connected the origin with the distal point, with a positive proximal direction, and the z-axis was perpendicular to the other two, with a positive direction to the right.


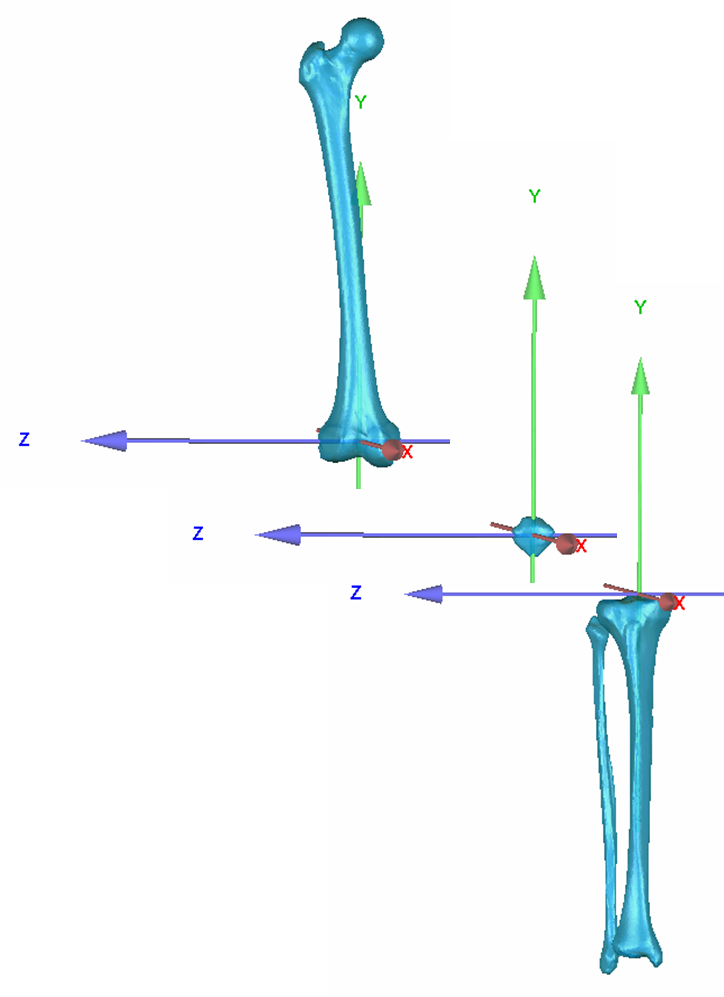


Figure S1_1: Anatomical reference systems for femur, patella and tibia

The segmentations of the leg, both pre- and post-preparation, were registered using an automatic algorithm in GeoMagic Studio software. This approach was also applied to align the digital reconstruction of the tibial and femoral bone surfaces, reference screws and patellar support (since the patella is internal to the joint capsule) digitalized through the optoelectronic system within the experimental reference system.

At the end of this registration process, the relative position and orientation of the anatomical reference systems with respect to the optoelectronic trackers could be determined. From here, the absolute and relative bone kinematics were computed through matrix transformations performed in MATLAB. In particular, the relative motions of the patella with respect to the femur and of the femur with respect to the tibia were calculated. Motions were parametrized using the Grood & Suntay convention [2], both for the rotation and translation components. It is worth noting that in this convention, the anterior-posterior displacement and ab-adduction rotation are measured along the axis perpendicular to both the medial-lateral axis of the femur and to the proximal-distal axis of the patella (patellofemoral kinematics) or the femur (tibiofemoral kinematics). Medial-lateral displacement and flexion-extension rotation are measured along the medial-lateral axis of the femur or tibia. Proximal-distal displacement and internal-external rotation are measured along the proximal-distal axis of the patella or femur.

The 6 motion components of the PF joint (3 rotations and 3 translations) and the 5 motion components of the TF joint (2 rotations and 3 translations, excluding the flexion-extension) were all considered functions of the imposed knee flexion-extension. Once assessed that the hysteresis between flexion and extension was negligible, a single cubic spline was fitted on all the kinematic data points obtained from the three flexion-extension cycles performed during the tests.

For each specimen the test in neutral direction QV_ML_ and QV_AP_ and 20 N of QV_load_ was considered as a reference. This makes it possible to isolate the differences between the subjects from the effect of QV variations. The impact of QV intensity and direction variations was quantified through the differences induced with respect to the neutral kinematics. In this way, it was possible to compare the curves of the different specimens and assess which of the three controlled parameters (QV_load_, QV_ML_, and QV_AP_) is influencing the kinematics of the PF and TF joints and to what extent, independently from individual differences in neutral conditions.

## **SUPPLEMENTARY MATERIAL #2**

### **Reference kinematics**

#### Patellofemoral joint

The PF joint exhibited similar kinematics across specimens under the same quadriceps loading conditions (Table S2_1, Figure S2_1). The reference condition corresponded to neutral QV angles and 20 N force.

In this condition, the patella rotated around its medio-lateral axis from 8.3° ± 9.1° (median and standard deviation between all specimens) at full extension to 81.9° ± 6.9° at maximum flexion. The standard deviation at maximum flexion was the highest throughout the range of motion.

The patella rotated around its antero-posterior axis from -2.8° ± 7.6° valgus in full extension, to 13.4° ± 5.9° varus in full flexion. A maximum inter-specimen deviation of 7.8° was observed during motion.

The patellar tilt showed a similar trend for all specimens, but different values. Almost all specimens were internally tilted at maximum extension, during mid flexion they tended to tilt externally, and then returned close to the starting position at maximum flexion. At full flexion, all specimens exhibited internal tilt of 4.0° ± 6.7°. The peak internal tilt (8.3° ± 7.1°) occurred at mid-flexion (60–70°). There was higher variability among subjects, with a standard deviation of 7.4° at approximately 90° of flexion.

The anterior-posterior translation showed a consistent pattern across specimens. At full extension, the patella origin was -47.4 mm ± 2.6 mm anterior to the origin of the femur, shifting by approximately 15 mm during flexion. This translation was nearly linear at intermediate flexion angles, with a maximum standard deviation of 3.7 mm.

During flexion, the patella moved distally, transitioning from 22.5 ± 7.4 mm proximal at full extension to -1.0 ± 4.7 mm distal at full flexion. The maximum inter-specimen deviation during motion was at full extension.

For medial-lateral translation, the patella shifted from a medial position (0.4 mm ± 3.9 mm) at full extension to a lateral position (8.5 mm ± 3.5 mm) at full flexion. The deviation between specimens was almost constant throughout the flexion cycles

#### Tibiofemoral joint

For the TF joint, the kinematics are summarized in Table S2_2. Kinematics showed slight variations in the five motion components of the TF during flexion (Figure S2_2). Notably, all specimens showed similar trends, although with relative off-sets. Only for internal-external rotation did the trend vary among the specimens, although it showed an interesting point of convergence between the curves around 90° (standard deviation 0.3°) (Figure S2_3). Variations were observed at low flexion angles, with some specimens showing initial internal rotation and others initial external rotation. During the early flexion phase, the femur tended, in average, to rotate internally relative to the tibia. This initial internal rotation phase peaks around 15°. The angle of maximum internal-rotation was found to be highly variable among the subjects, with a standard deviation of about 6°. Thereafter, the curves showed a progressive transition, with a point of intersection around 90° of flexion, where the internal-external rotation was approximately 0° for all specimens.

The abduction-adduction curves showed wide variability among the specimens, with deviations of up to 7.7° along the entire range of motion. The general trend suggested initial abduction followed by progressive adduction as flexion increases.

Table S2_1: Patellofemoral joint: Components of motion (Rotations and translations) for all the specimens in the reference condition. The values were calculated at 30° intervals of the knee flexion angles, for patella vs femur motion. The median and standard deviation between 12 specimens is reported.

| Knee flexion | 0° | 30° | 60° | 90° | 120° |
| --- | --- | --- | --- | --- | --- |
| Flexion(+)/Extension(-)[°] | 8.34 ± 9.14 | 24.59 ± 4.78 | 44.47 ± 5.21 | 65.35 ± 6.11 | 81.90 ± 6.92 |
| Valgus(+)/Varus(-)[°] | -2.81 ± 7.62 | -0.63 ± 7.76 | 4.52 ± 4.88 | 11.16 ± 3.50 | 13.36 ± 5.94 |
| Internal(+)/External(-) tilt[°] | 4.00 ± 6.71 | 6.20 ± 4.95 | 8.26 ± 7.08 | 7.08 ± 7.45 | 3.79 ± 5.13 |
| Anterior(+)/Posterior(-) translation [mm] | -47.38 ± 2.58 | -47.00 ± 2.10 | -41.86±2.48 | -36.24 ± 3.15 | -31.90 ± 3.67 |
| Proximal(+)/Distal(-) translation [mm] | 22.53 ± 7.41 | 12.19 ± 5.81 | 5.99 ± 5.42 | 3.18 ± 4.69 | -0.97 ± 4.67 |
| Lateral(+)/Medial(-) translation [mm] | 0.41 ± 3.87 | 0.47 ± 3.87 | 4.26 ± 3.13 | 8.42 ± 3.07 | 8.52 ± 3.54 |

Table S2_2: Tibiofemoral joint: Components of motion (Rotations and translations) for all the specimens in the reference condition. The values were calculated at 30° intervals of the knee flexion angles, for patella vs femur motion. The median and standard deviation between 12 specimens is reported.

| Knee flexion | 0° | 30° | 60° | 90° | 120° |
| --- | --- | --- | --- | --- | --- |
| Abduction(+)/Adduction(-)[°] | 1.99 ± 5.29 | 4.65 ± 7.49 | 4.70 ± 7.71 | 4.84 ± 7.05 | 2.57 ± 6.74 |
| Internal(+)/External(-) rotation[°] | -0.65 ± 5.83 | 2.14 ± 5.59 | 1.85 ± 2.96 | -0.13 ± 0.31 | -2.01 ± 2.32 |
| Anterior(+)/Posterior(-) translation [mm] | -3.06 ± 3.47 | -1.17 ± 3.60 | 3.51 ± 3.85 | 9.20 ± 4.56 | 14.30 ± 5.50 |
| Proximal(+)/Distal(-) translation [mm] | 22.78 ± 2.91 | 23.86 ± 2.58 | 25.64 ± 2.30 | 25.18 ± 1.66 | 23.45 ± 1.84 |
| Lateral(+)/Medial(-) translation [mm] | -4.01 ± 3.67 | -2.87 ± 3.56 | -2.01 ± 3.76 | -1.61 ± 3.75 | 1.33 ± 3.62 |


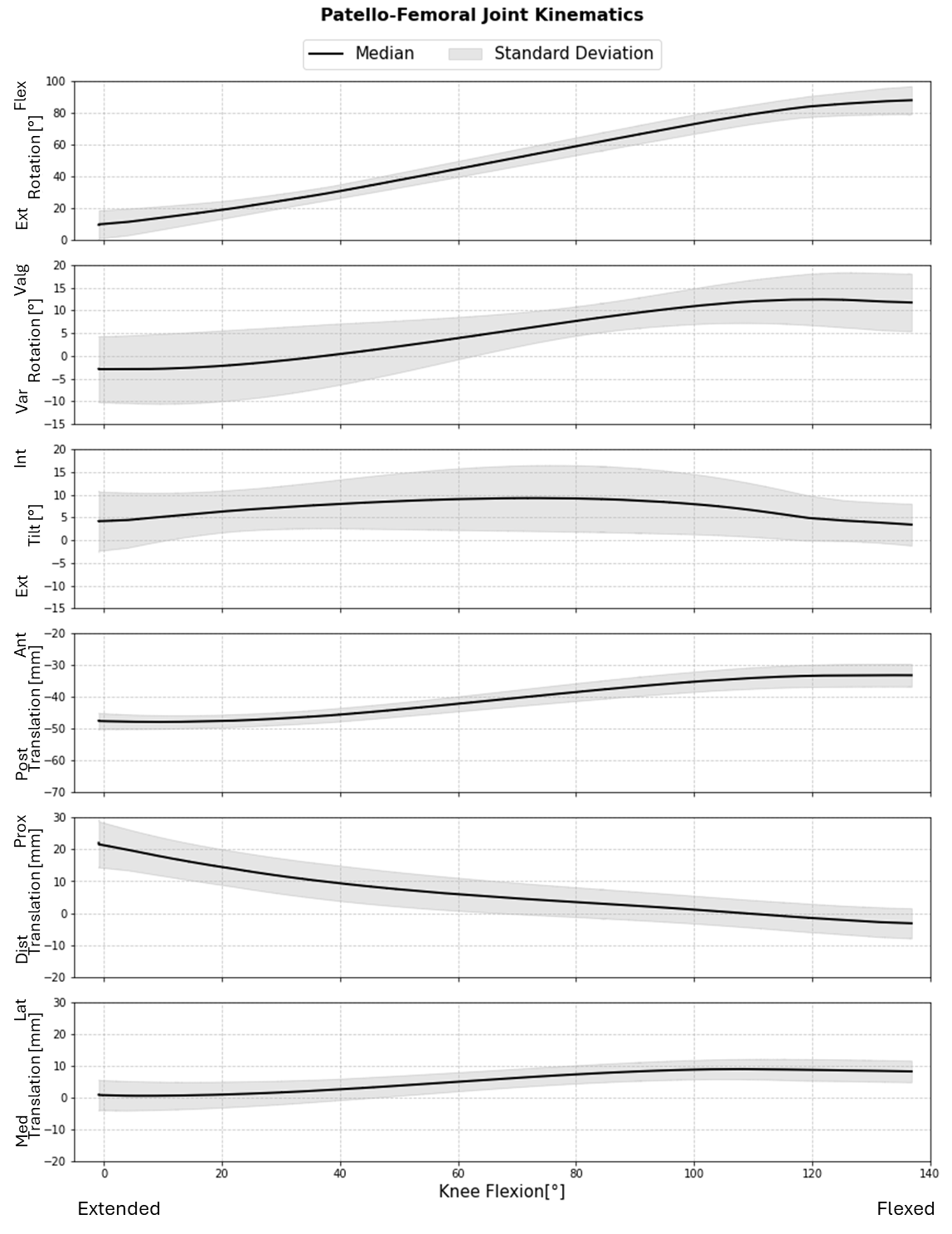


Figure S2_1: Patellofemoral joint: Kinematics in the reference condition (neutral direction and 20N of load): median values (black line) and standard deviation (grey shaded area) for flexion/extension, varus/valgus, internal/external tilt angles, and anterior-posterior, proximal-distal, and medial-lateral translations. The data are plotted as a function of knee flexion angle, representing the motion of the patella with respect to the femur.


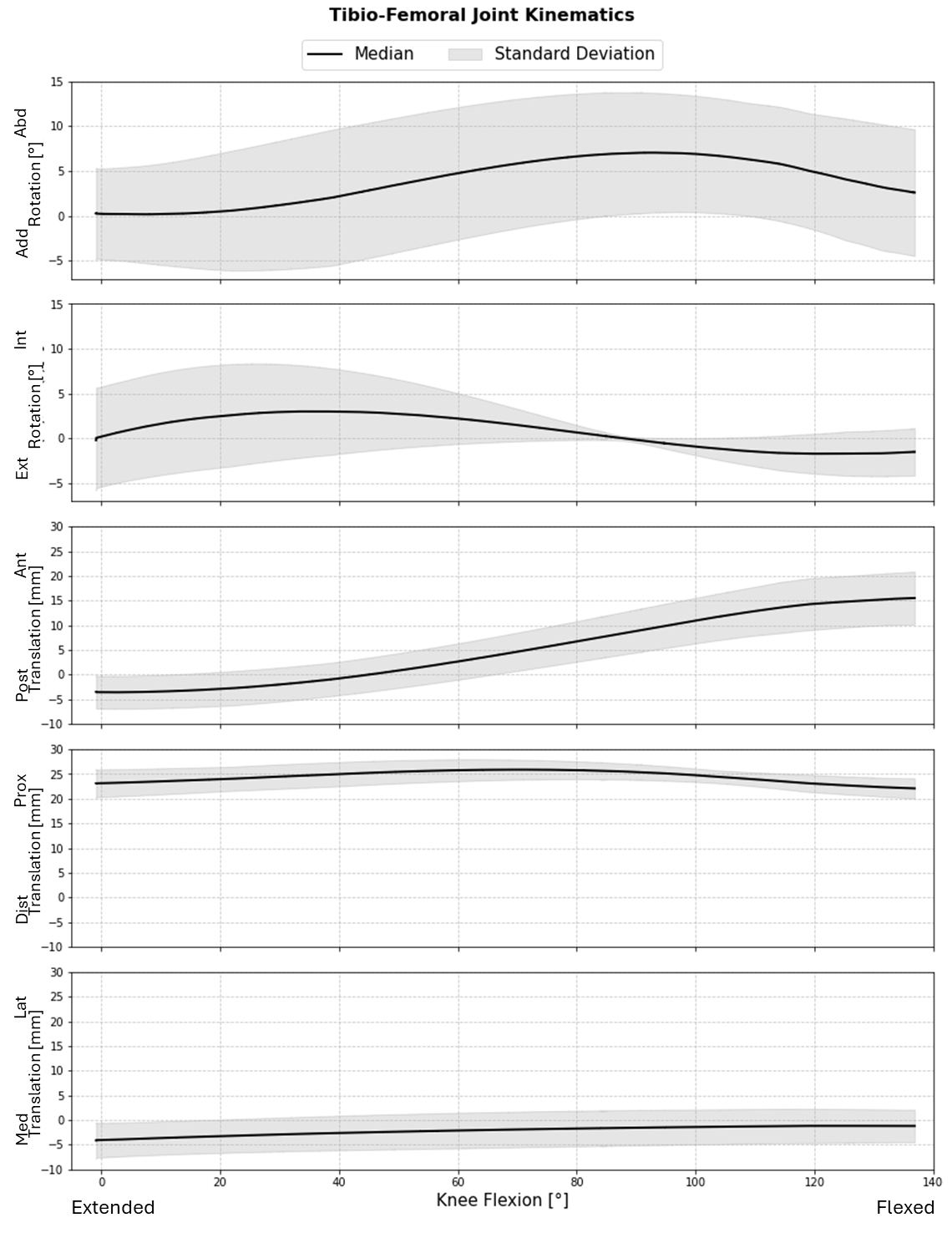
Figure S2_2: Tibiofemoral joint: Kinematics in the reference condition (neutral direction and 20N of load): median values (black line) and standard deviation (grey shaded area) for flexion/extension, varus/valgus, internal/external tilt angles, and anterior-posterior, proximal-distal, and medial-lateral translations. The data are plotted as a function of knee flexion angle, representing the motion of the patella with respect to the femur.


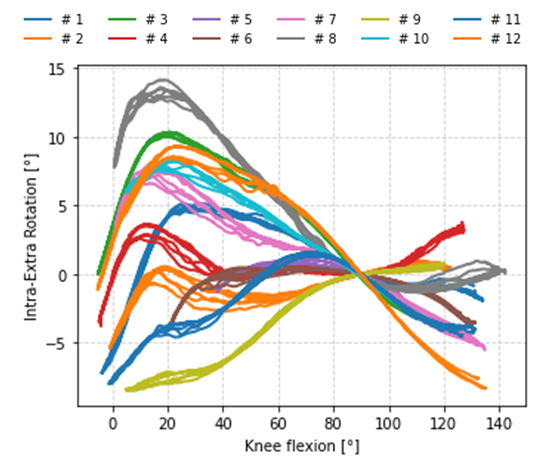


Figure S2_3: Intra-Extra Rotation of tibiofemoral joint for all specimens as a function of knee flexion angle.

## **SUPPLEMENTARY MATERIAL #3**

### **Influence of the quadriceps muscle for all degrees of freedom of the patellofemoral joint**

In the main article only the most influent patellar motion were presented, in this section of the supplementary material, we report all the degrees of freedom of the patellofemoral joint with the mean and standard deviation of the differences in patellofemoral kinematics resulting from variations in quadriceps load. The methodology used to compute these values is described in the main article and summarized here: the curves of flexion/extension, varus/valgus rotation, internal/external tilt, and translations along the three anatomical axes of the patella (Figure S3_1) with respect to the femur were interpolated using a cubic spline as a function of the knee flexion angle.

The kinematics of a representative specimen, with curves grouped according to the three parameters considered for quadriceps load variation—medial-lateral direction (QV-ML), load magnitude (QV-load), and anterior-posterior direction (QV-AP)—are shown in Figures S3_2 to S3_7.

To assess the influence of quadriceps force variation across all specimens, the reference test values (QV-ML = neutral, QV-load = 20N, QV-AP = neutral) were subtracted from each specimen’s data. The mean and standard deviation of the differences in patellofemoral kinematics are presented in Figures S3_8 to S3_13, with curves grouped by quadriceps parameter. Each graph also reports the p-value, indicating the statistical significance of the effect of that parameter on patellar motion.


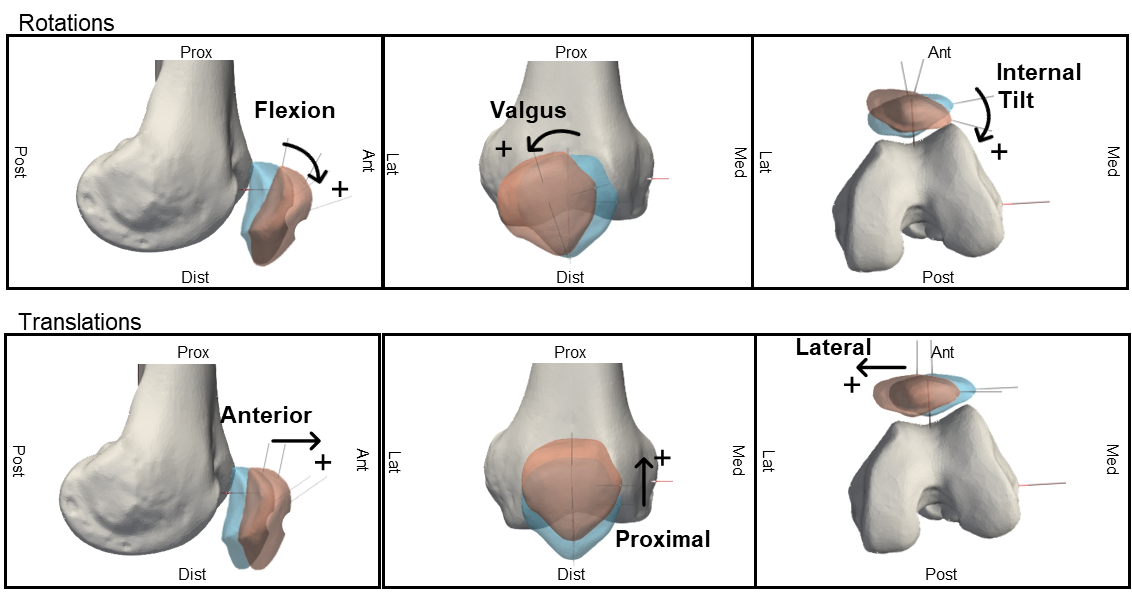
Figure S3_1 Degrees of freedom of the patellofemoral joint


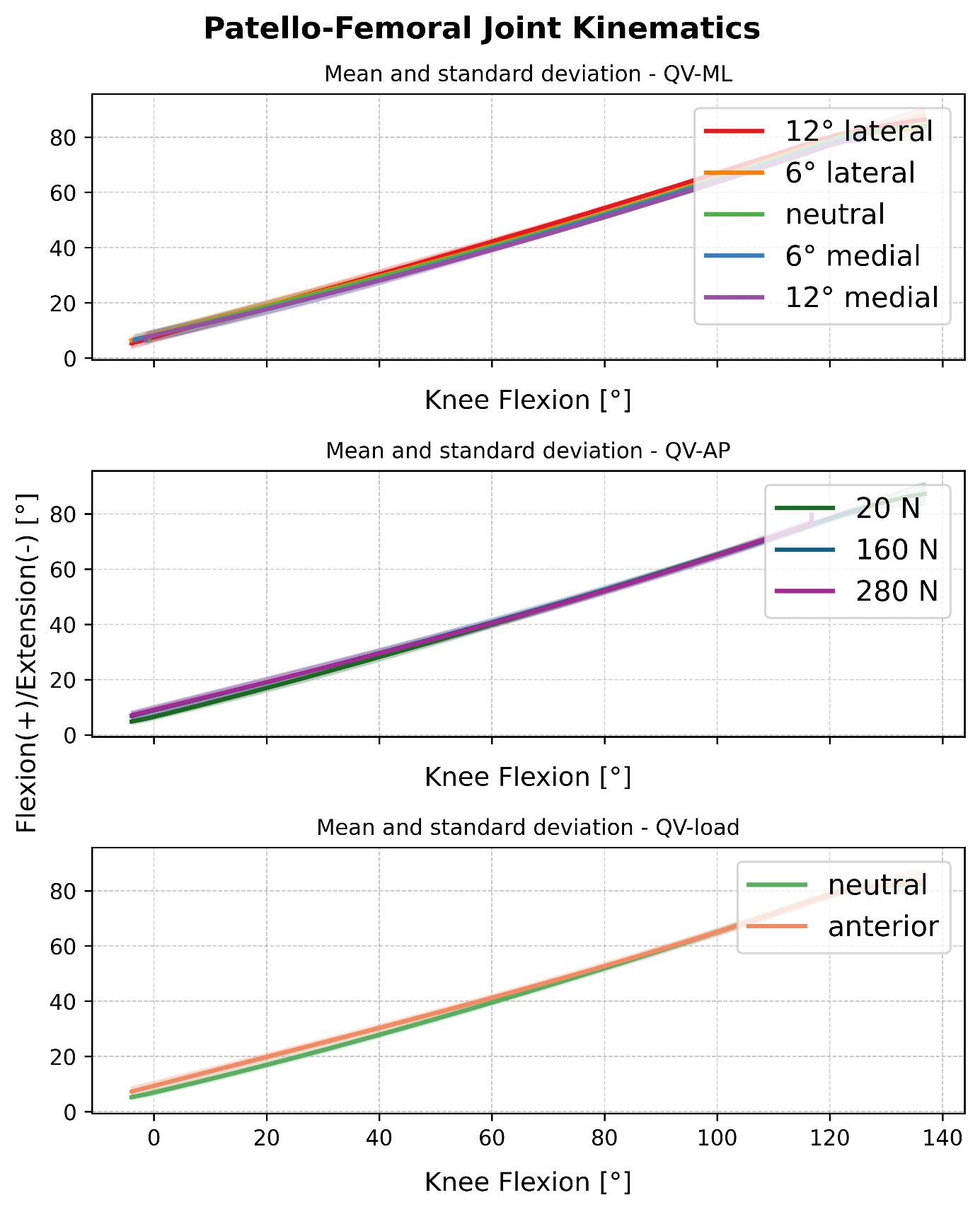


Figure S3_2 Flexion/Extension rotation of one specimen (#1) with respect to the knee flexion for all the tests, grouped by QV-ML in the top, QV-load in the middle and QV-AP in the bottom


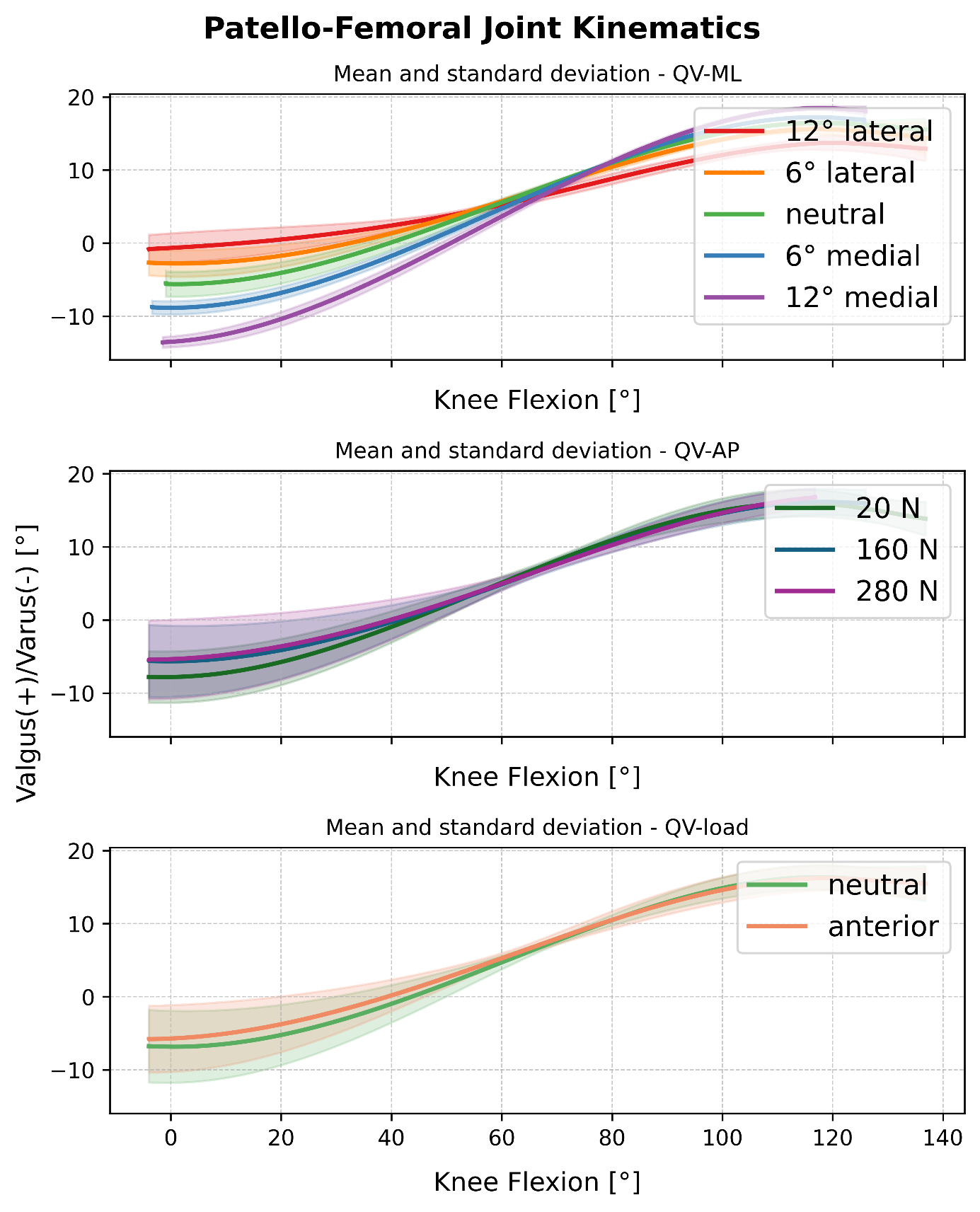
 Figure S3_3 Valgus/varus rotation of one specimen (#1) with respect to the knee flexion for all the tests, grouped by QV-ML in the top, QV-load in the middle and QV-AP in the bottom


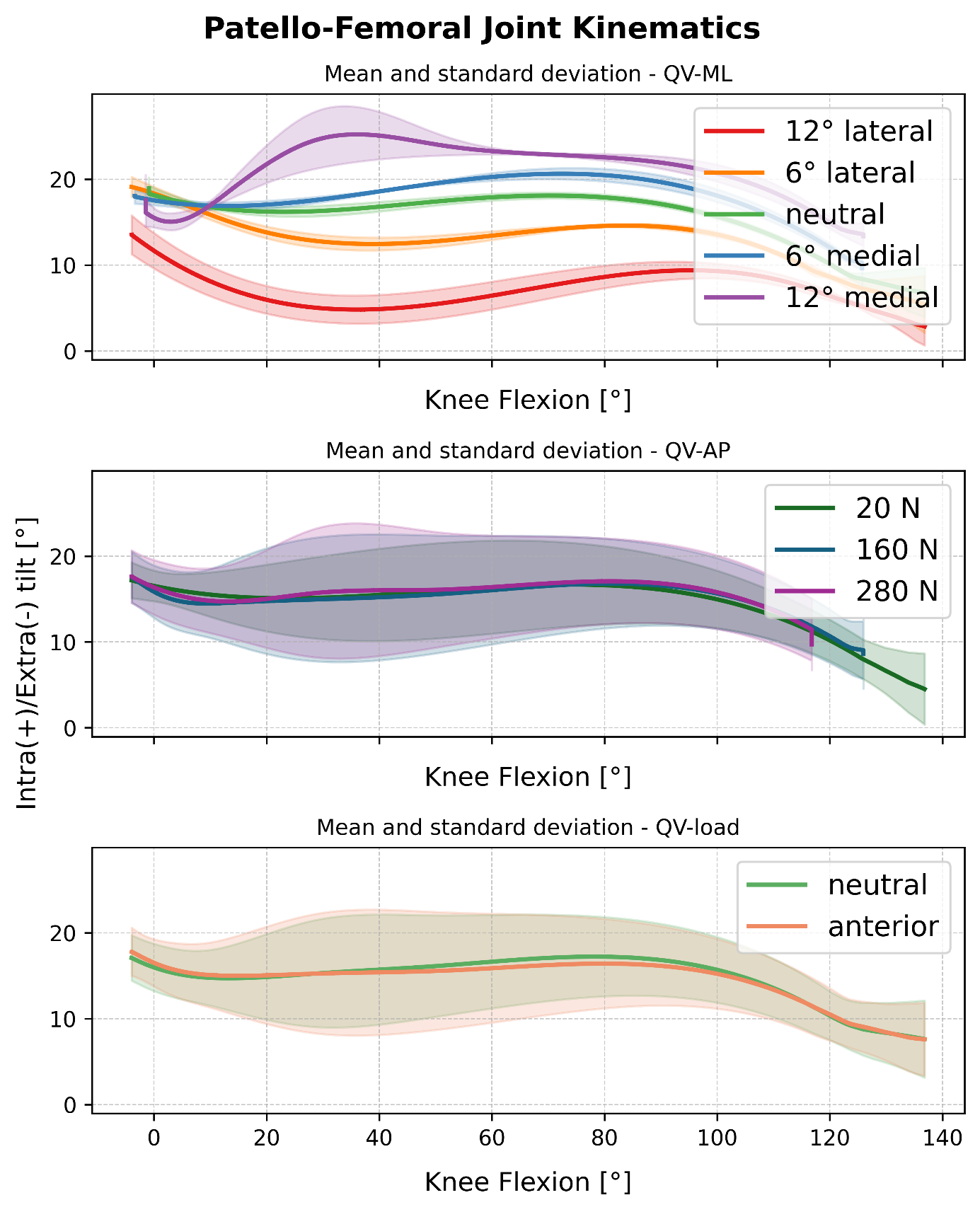
Figure S3_4 Internal/external tilt of one specimen (#1) with respect to the knee flexion for all the tests, grouped by QV-ML in the top, QV-load in the middle and QV-AP in the bottom


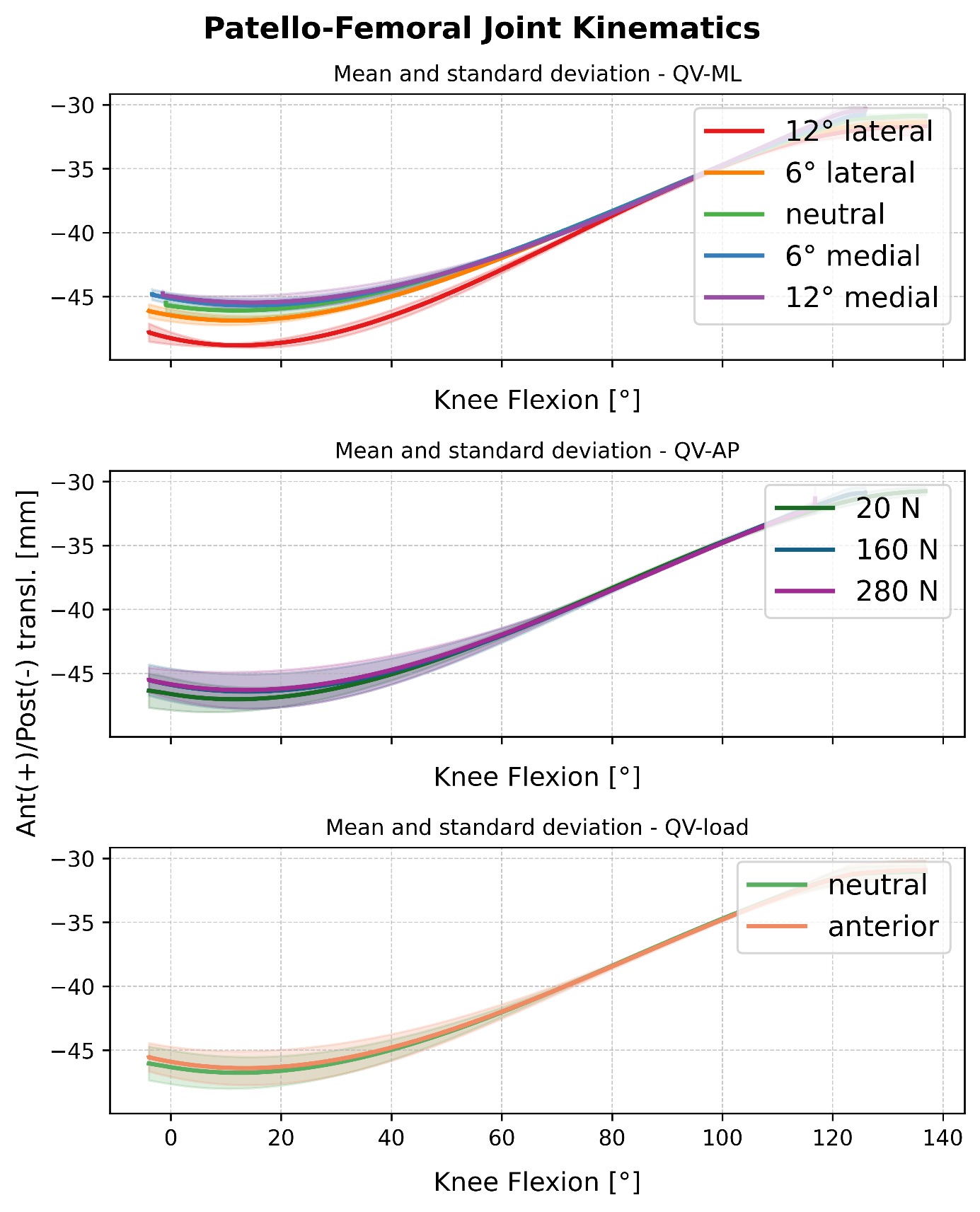
Figure S3_5 Anterior/posterior translation of one specimen (#1) with respect to the knee flexion for all the tests, grouped by QV-ML in the top, QV-load in the middle and QV-AP in the bottom


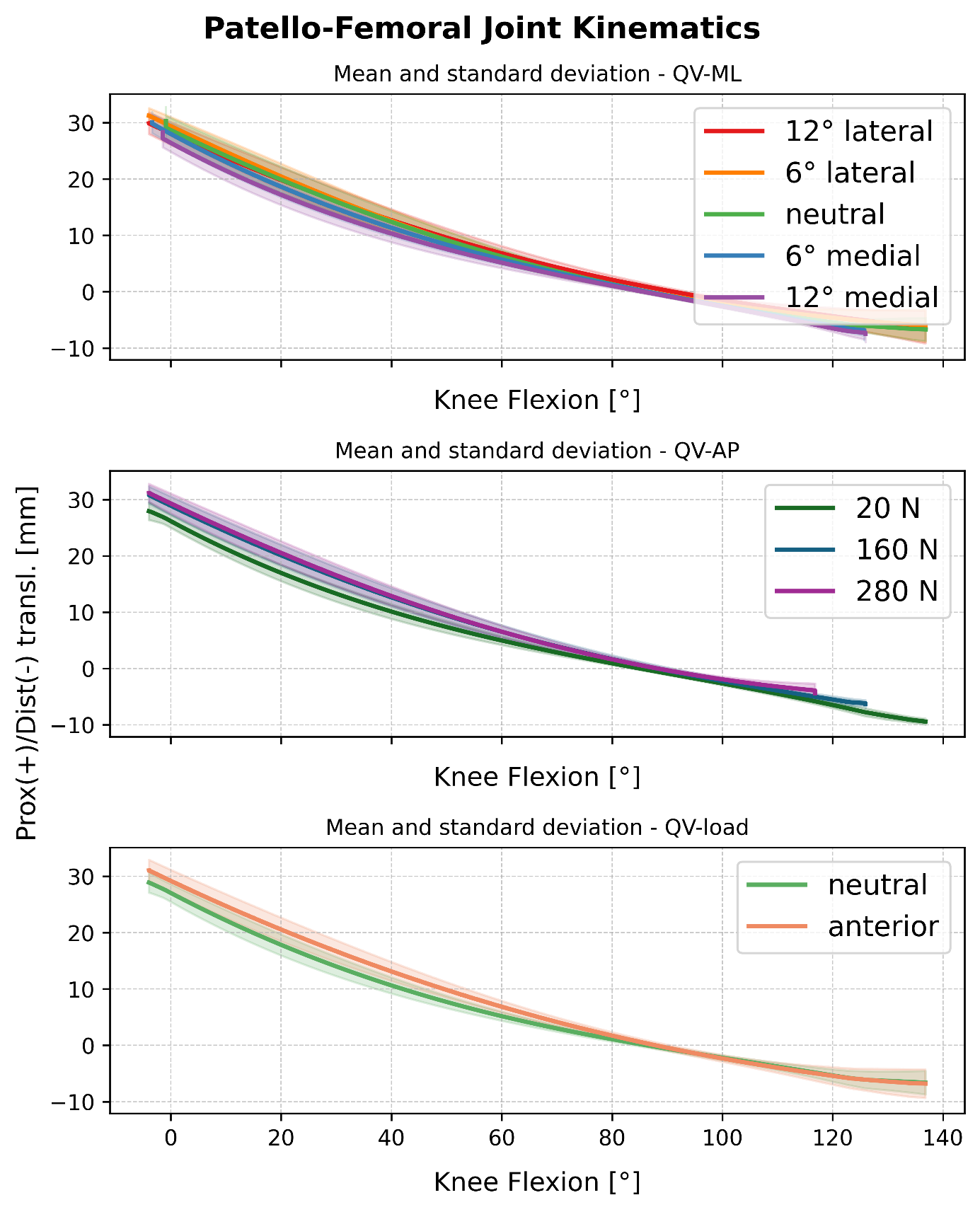
Figure S3_6 Proximal/distal translation of one specimen (#1) with respect to the knee flexion for all the tests, grouped by QV-ML in the top, QV-load in the middle and QV-AP in the bottom


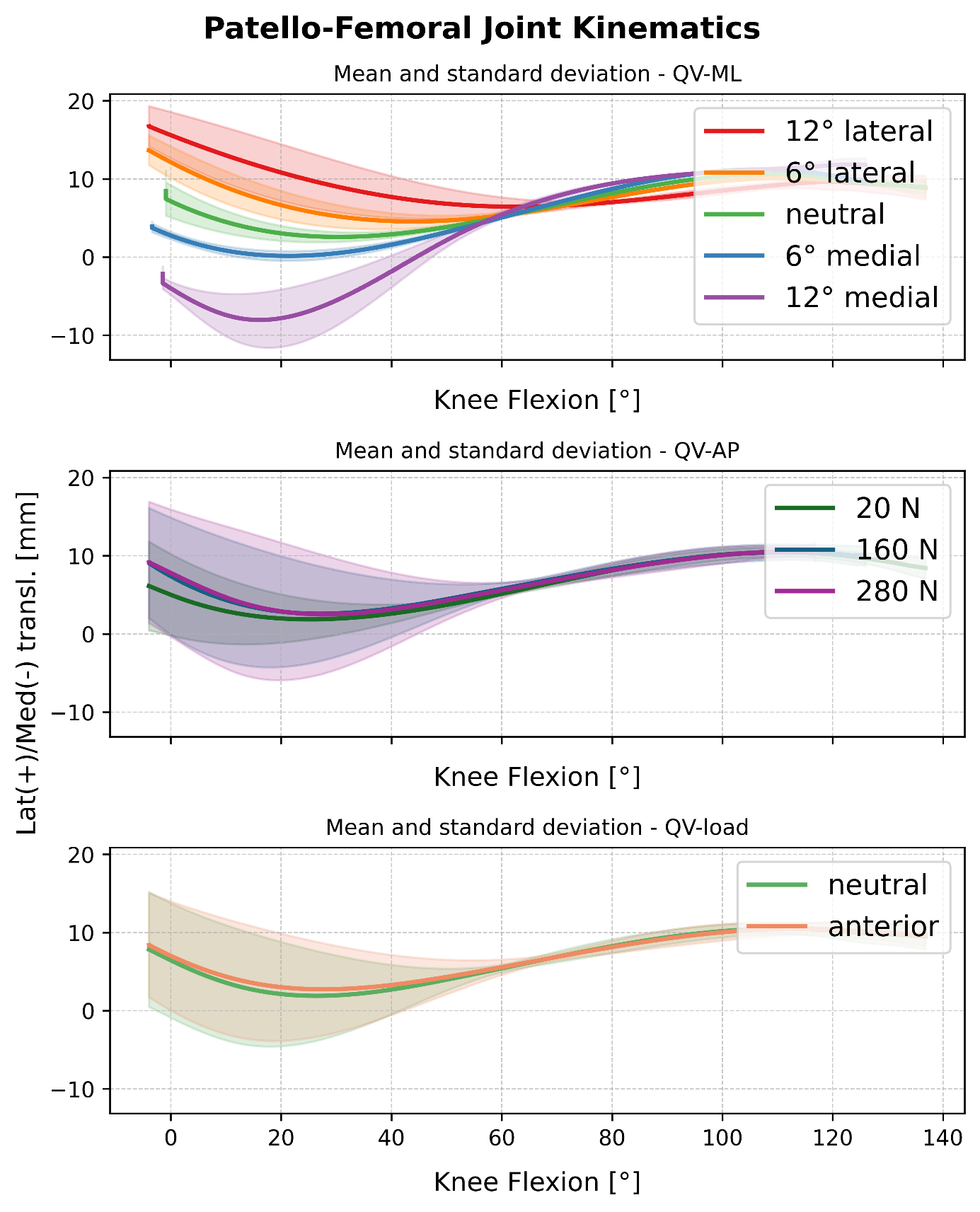
 Figure S3_7 Lateral/medial translation of one specimen (#1) with respect to the knee flexion for all the tests, grouped by QV-ML in the top, QV-load in the middle and QV-AP in the bottom


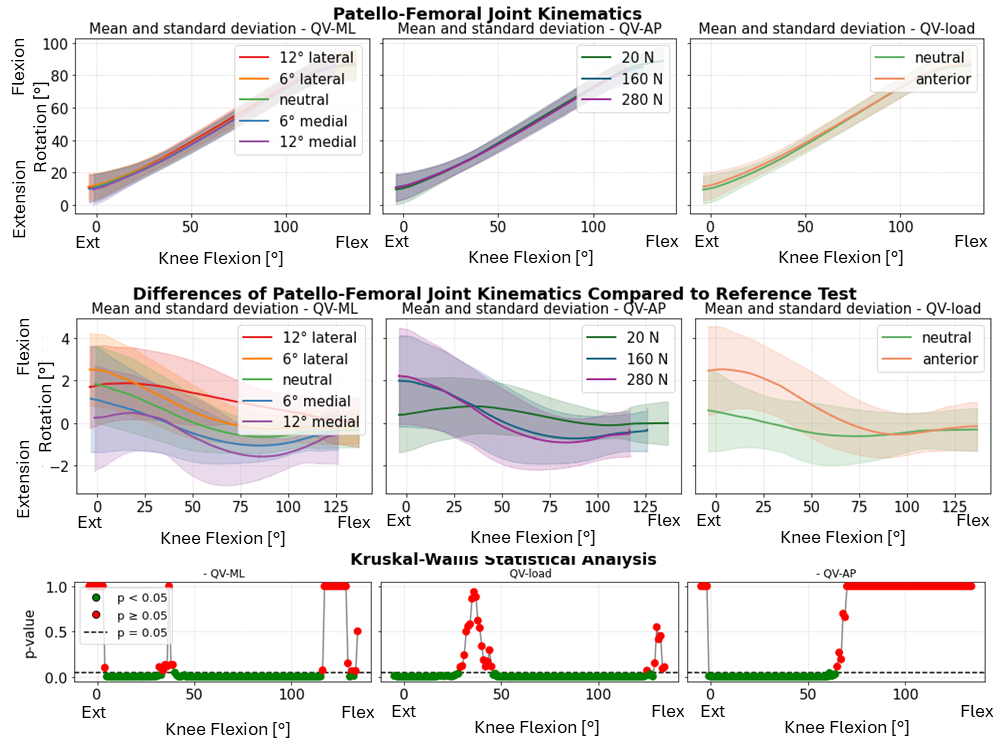


Figure S3_8: Flexion-Extension rotation of the patella. TOP: absolute values as a function of knee flexion angle (the median and standard deviation between 12 specimens are plotted). CENTER: differences of all tests compared with the reference test (QVload = 20 N, QVML = neutral, QVAP = posterior). Left shows the difference as a function of QVML, middle QVload, right QVAP. BOTTOM: significance of the differences plotted at the center. The p-value trend is plotted for the three parameters (left QVML, middle QVload, right QVAP); the significant values are highlighted in green (p<0.05), the non significant ones in red (p≥0.05).


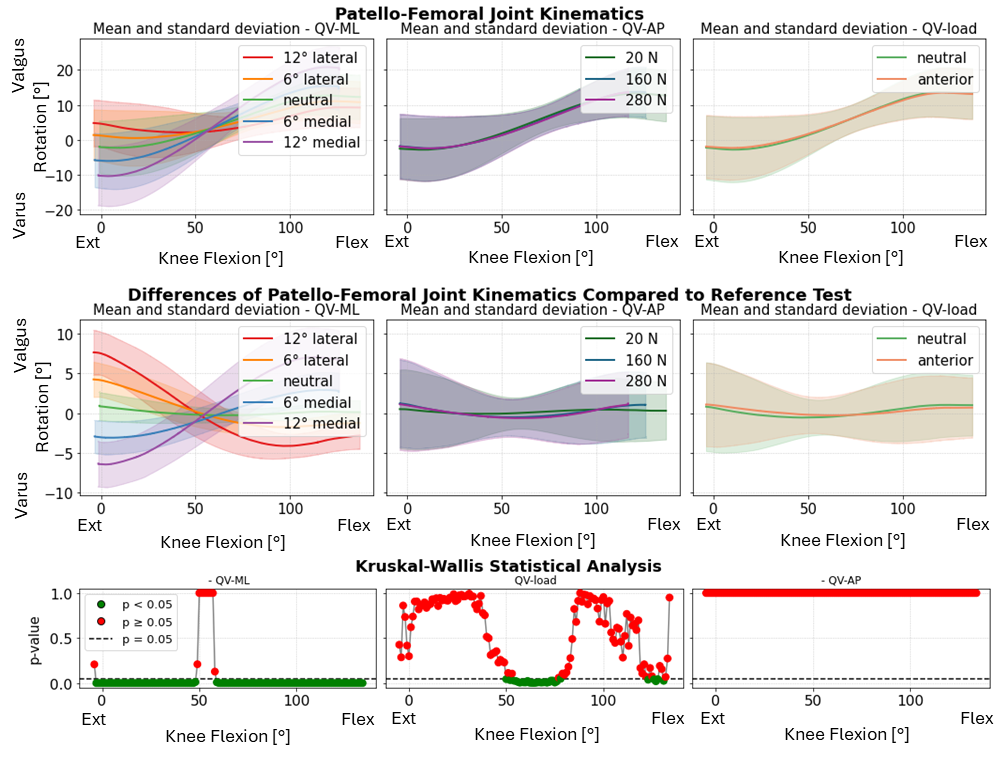


Figure S3_9: Varus-valgus rotation of the patella. TOP: absolute values as a function of knee flexion angle (the median and standard deviation between 12 specimens are plotted). CENTER: differences of all tests compared with the reference test (QVload = 20 N, QVML = neutral, QVAP = posterior). Left shows the difference as a function of QVML, middle QVload, right QVAP. BOTTOM: significance of the differences plotted at the center. The p-value trend is plotted for the three parameters (left QVML, middle QVload, right QVAP); the significant values are highlighted in green (p<0.05), the non significant ones in red (p≥0.05).


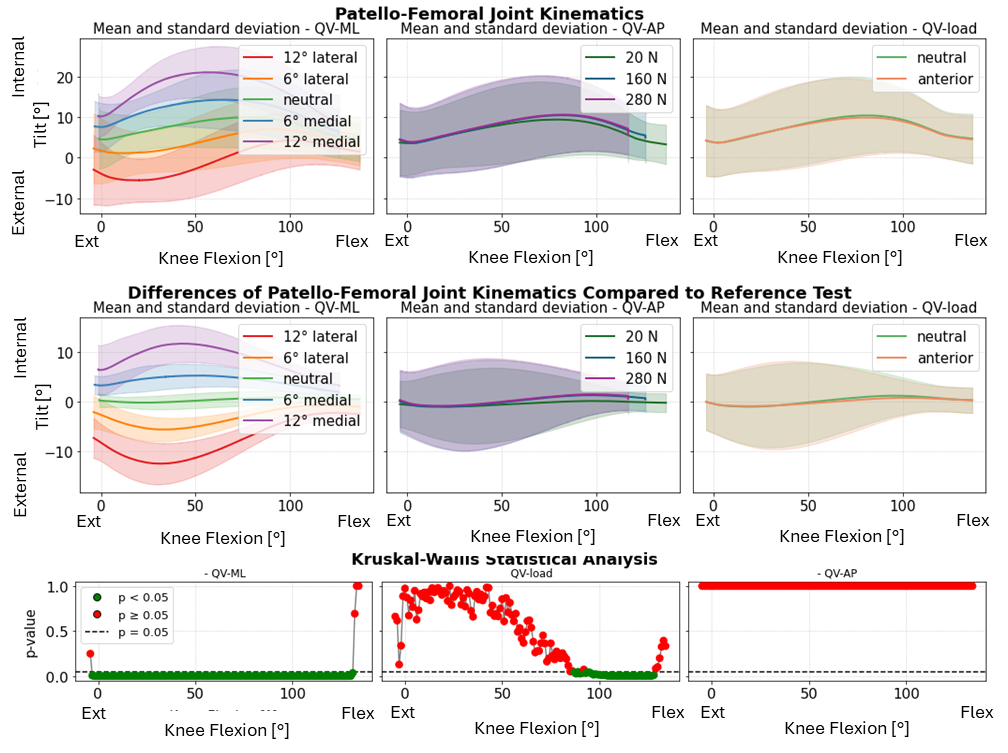


Figure S3_10: Internal-External of the patella. TOP: absolute values as a function of knee flexion angle (the median and standard deviation between 12 specimens are plotted). CENTER: differences of all tests compared with the reference test (QVload = 20 N, QVML = neutral, QVAP = posterior). Left shows the difference as a function of QVML, middle QVload, right QVAP. BOTTOM: significance of the differences plotted at the center. The p-value trend is plotted for the three parameters (left QVML, middle QVload, right QVAP); the significant values are highlighted in green (p<0.05), the non significant ones in red (p≥0.05).


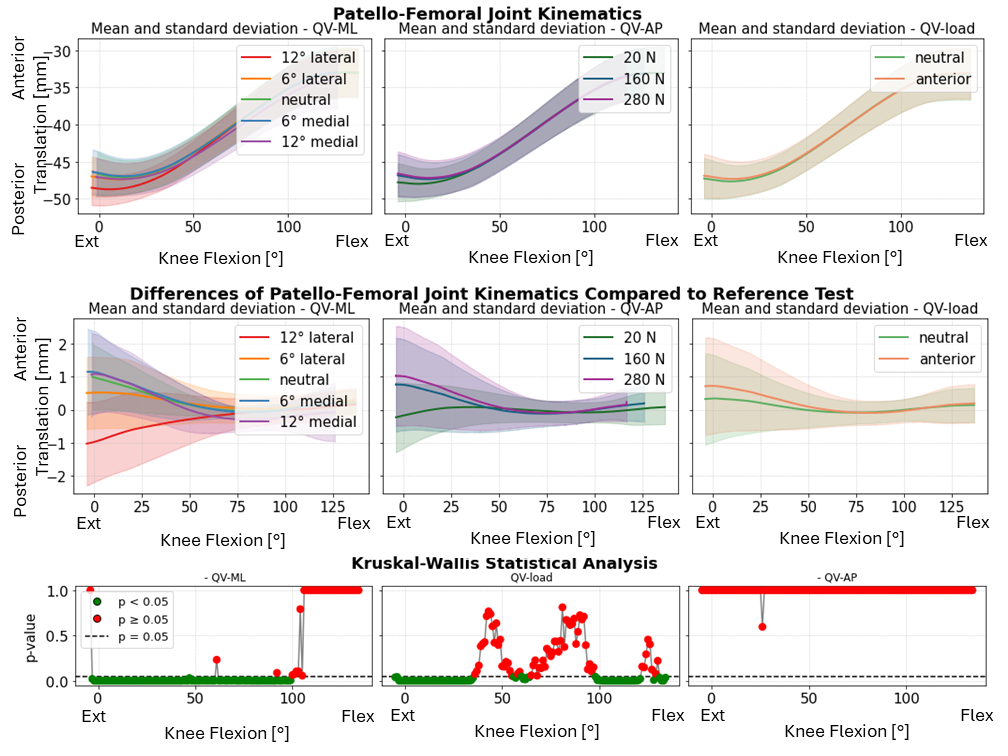


Figure S3_11: Anterior-Posterior translation of the patella. TOP: absolute values as a function of knee flexion angle (the median and standard deviation between 12 specimens are plotted). CENTER: differences of all tests compared with the reference test (QVload = 20 N, QVML = neutral, QVAP = posterior). Left shows the difference as a function of QVML, middle QVload, right QVAP. BOTTOM: significance of the differences plotted at the center. The p-value trend is plotted for the three parameters (left QVML, middle QVload, right QVAP); the significant values are highlighted in green (p<0.05), the non significant ones in red (p≥0.05).


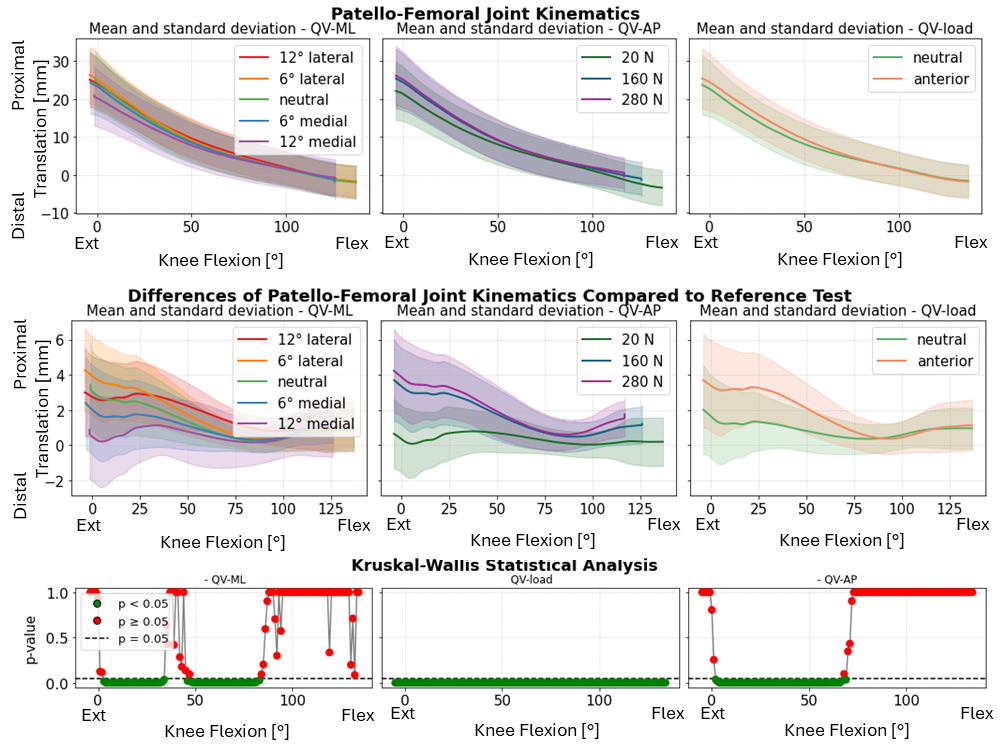


Figure S3_12: Proximal-Distal translation of the patella. TOP: absolute values as a function of knee flexion angle (the median and standard deviation between 12 specimens are plotted). CENTER: differences of all tests compared with the reference test (QVload = 20 N, QVML = neutral, QVAP = posterior). Left shows the difference as a function of QVML, middle QVload, right QVAP. BOTTOM: significance of the differences plotted at the center. The p-value trend is plotted for the three parameters (left QVML, middle QVload, right QVAP); the significant values are highlighted in green (p<0.05), the non significant ones in red (p≥0.05).


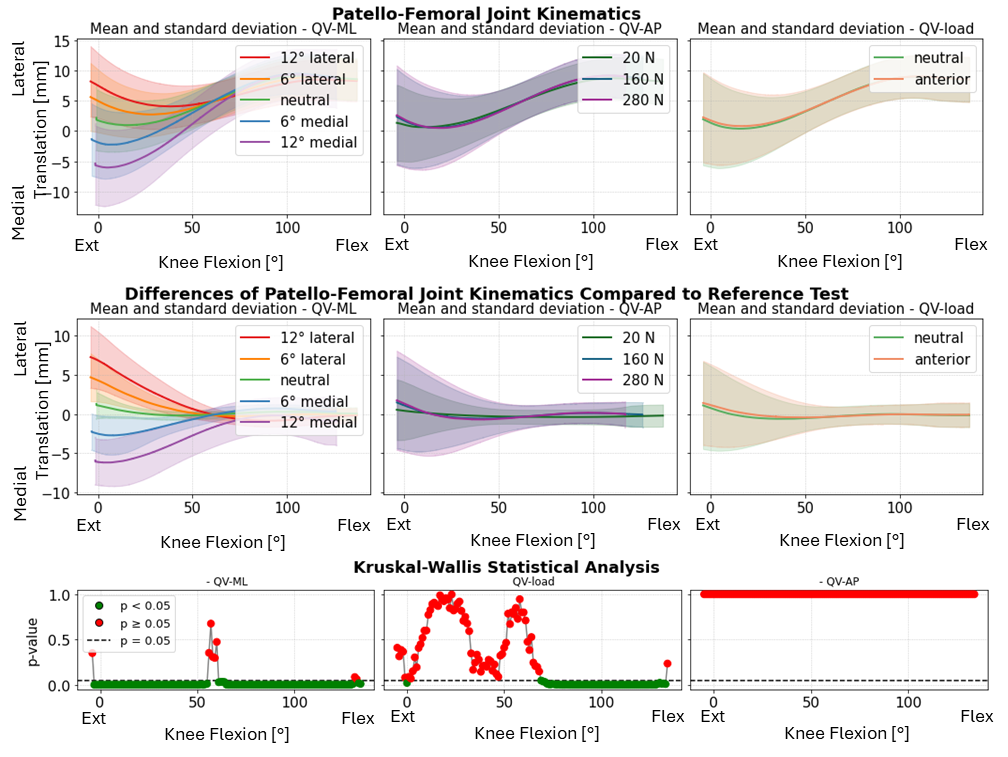


Figure S3_13: Medial-Lateral translation of the patella. TOP: absolute values as a function of knee flexion angle (the median and standard deviation between 12 specimens are plotted). CENTER: differences of all tests compared with the reference test (QVload = 20 N, QVML = neutral, QVAP = posterior). Left shows the difference as a function of QVML, middle QVload, right QVAP. BOTTOM: significance of the differences plotted at the center. The p-value trend is plotted for the three parameters (left QVML, middle QVload, right QVAP); the significant values are highlighted in green (p<0.05), the non significant ones in red (p≥0.05).

## **SUPPLEMENTARY MATERIAL #4**

### **Influence of the quadriceps muscle for all degrees of freedom of the tibiofemoral joint**

In this section of the supplementary material, we report the motion of the femur with respect to the tibia in all its degrees of freedom (Figure S3_1).

The kinematics of a representative specimen, with curves grouped according to the three parameters considered for quadriceps load variation—medial-lateral direction (QV-ML), load magnitude (QV-load), and anterior-posterior direction (QV-AP)—are shown in Figures S4_2 to S4_6.

The mean and standard deviation of the differences in tibiofemoral kinematics with respect to the reference test are presented in Figures S4_7 to S4_11, with curves grouped by quadriceps parameter. Each graph also reports the p-value, indicating the statistical significance of the effect of that parameter on patellar motion


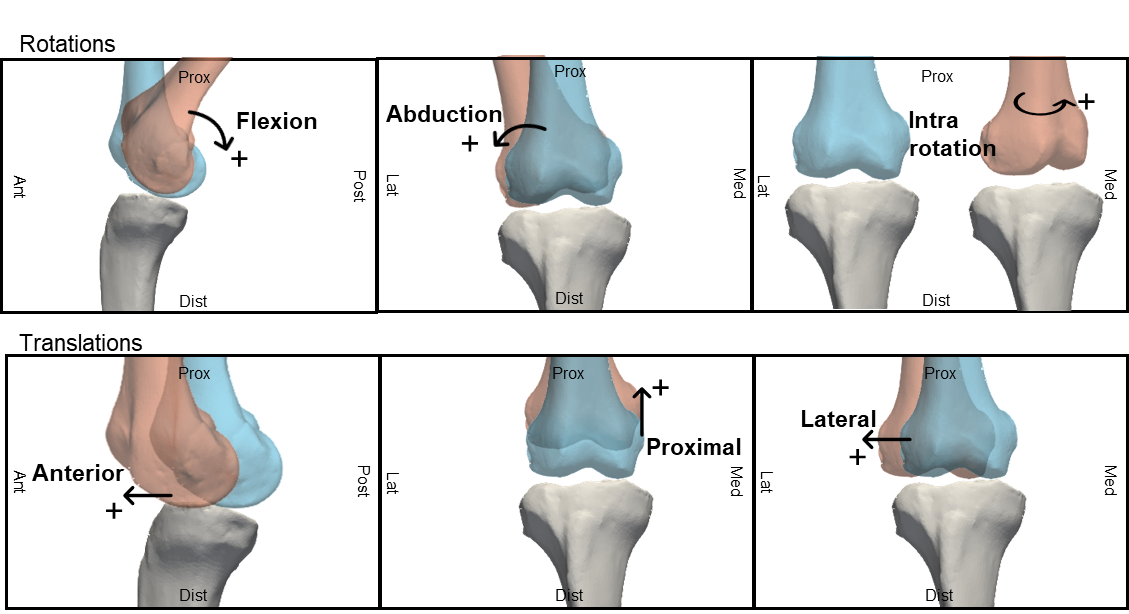


Figure S4_1 Degrees of freedom of the tibiofemoral joint. Figshare repository (https://doi.org/10.6084/m9.figshare.29278721).


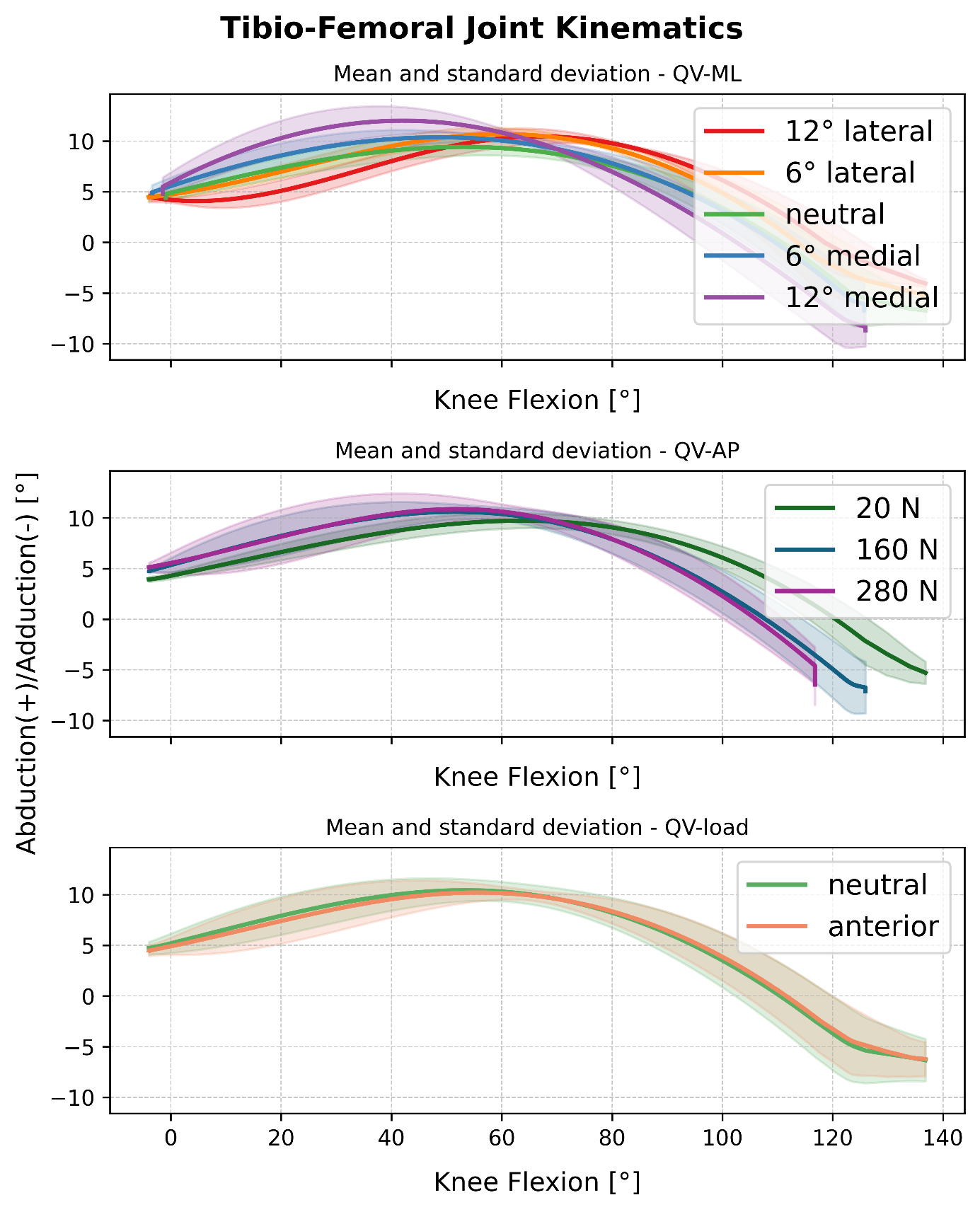
 Figure S4_2 Abduction/adduction rotation of one specimen (#1) with respect to the knee flexion for all the tests, grouped by QV-ML in the top, QV-load in the middle and QV-AP in the bottom


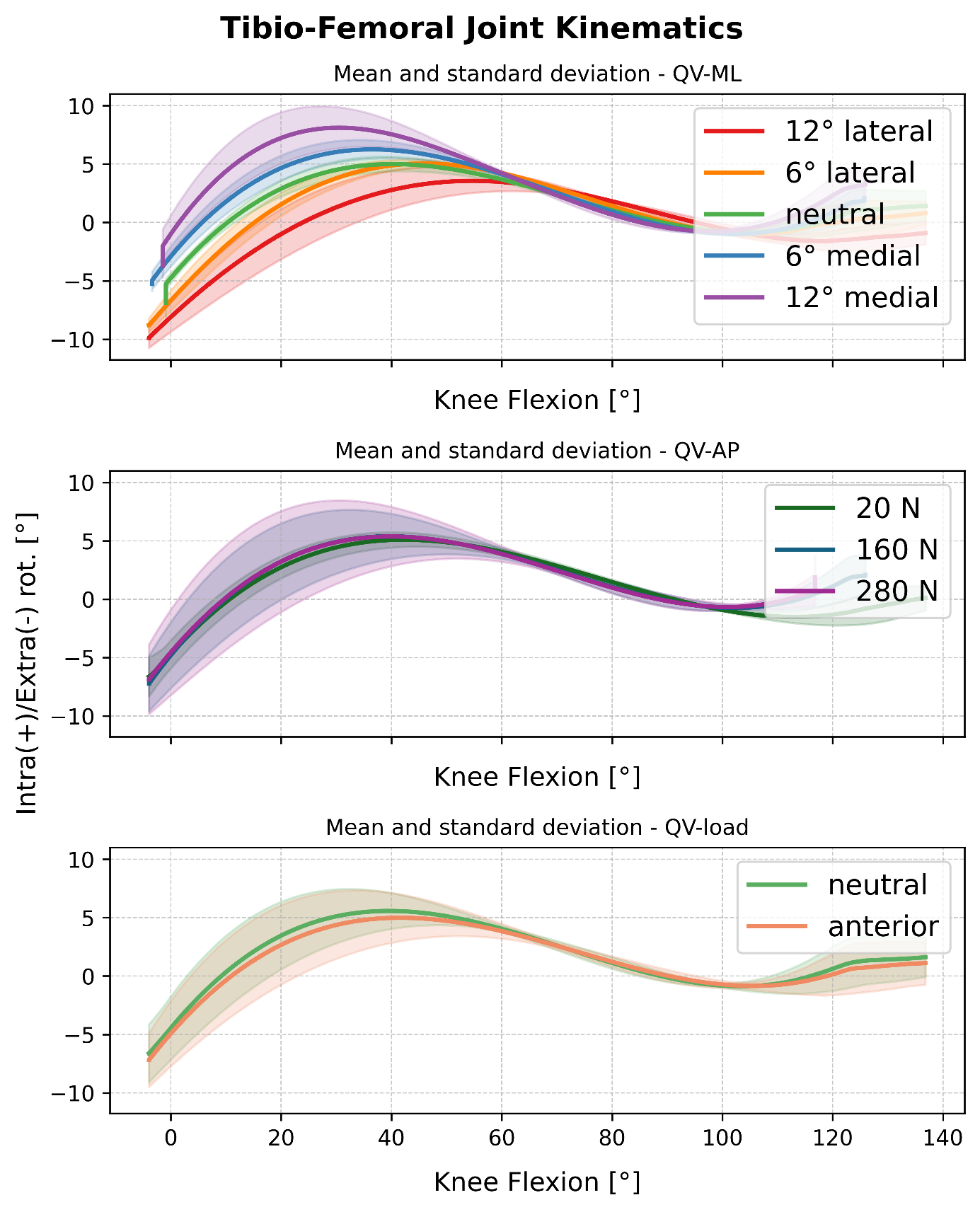
 Figure S4_3 Intra/extra rotation of one specimen (#1) with respect to the knee flexion for all the tests, grouped by QV-ML in the top, QV-load in the middle and QV-AP in the bottom


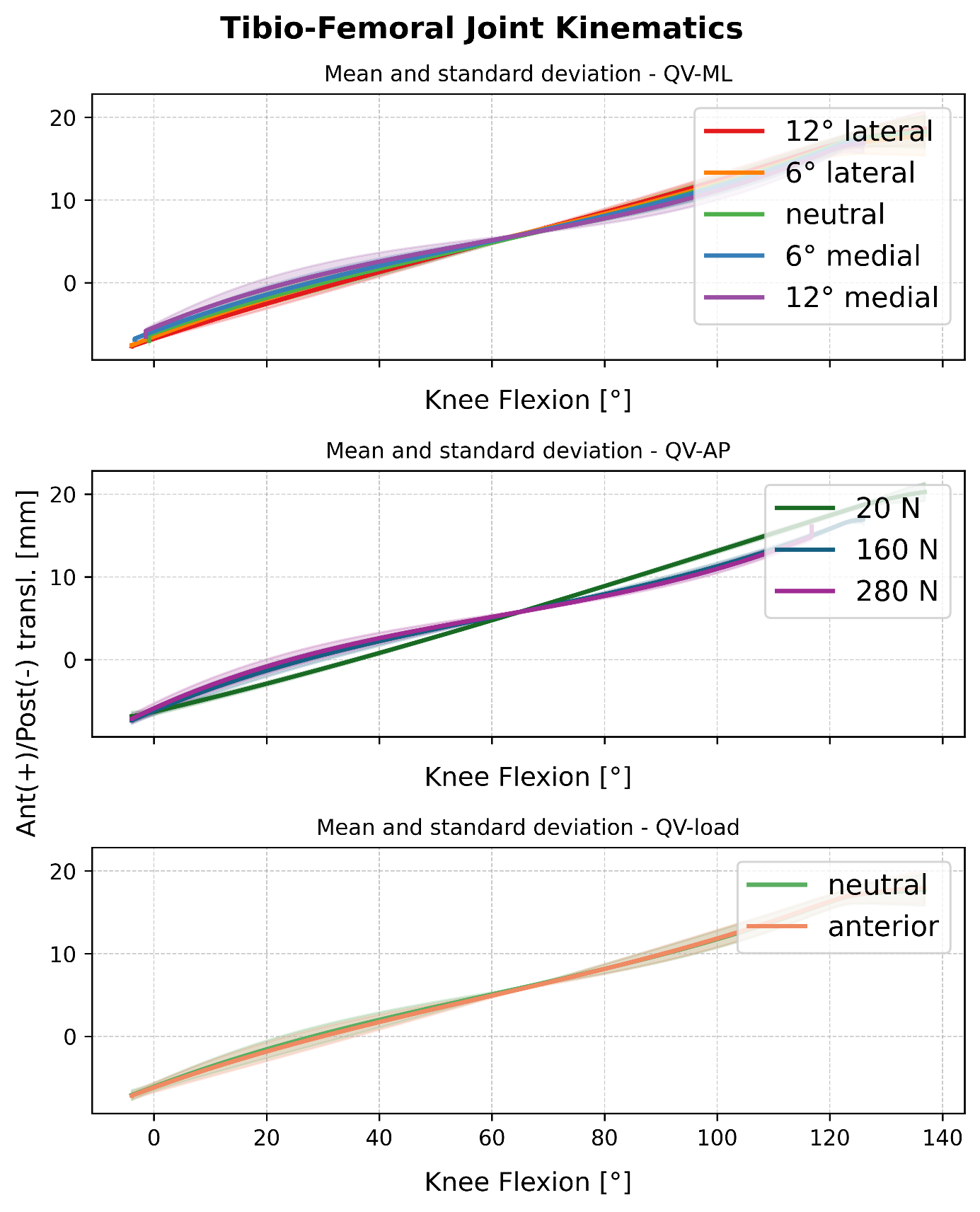
 Figure S4_4 Anterior/posterior translation of one specimen (#1) with respect to the knee flexion for all the tests, grouped by QV-ML in the top, QV-load in the middle and QV-AP in the bottom


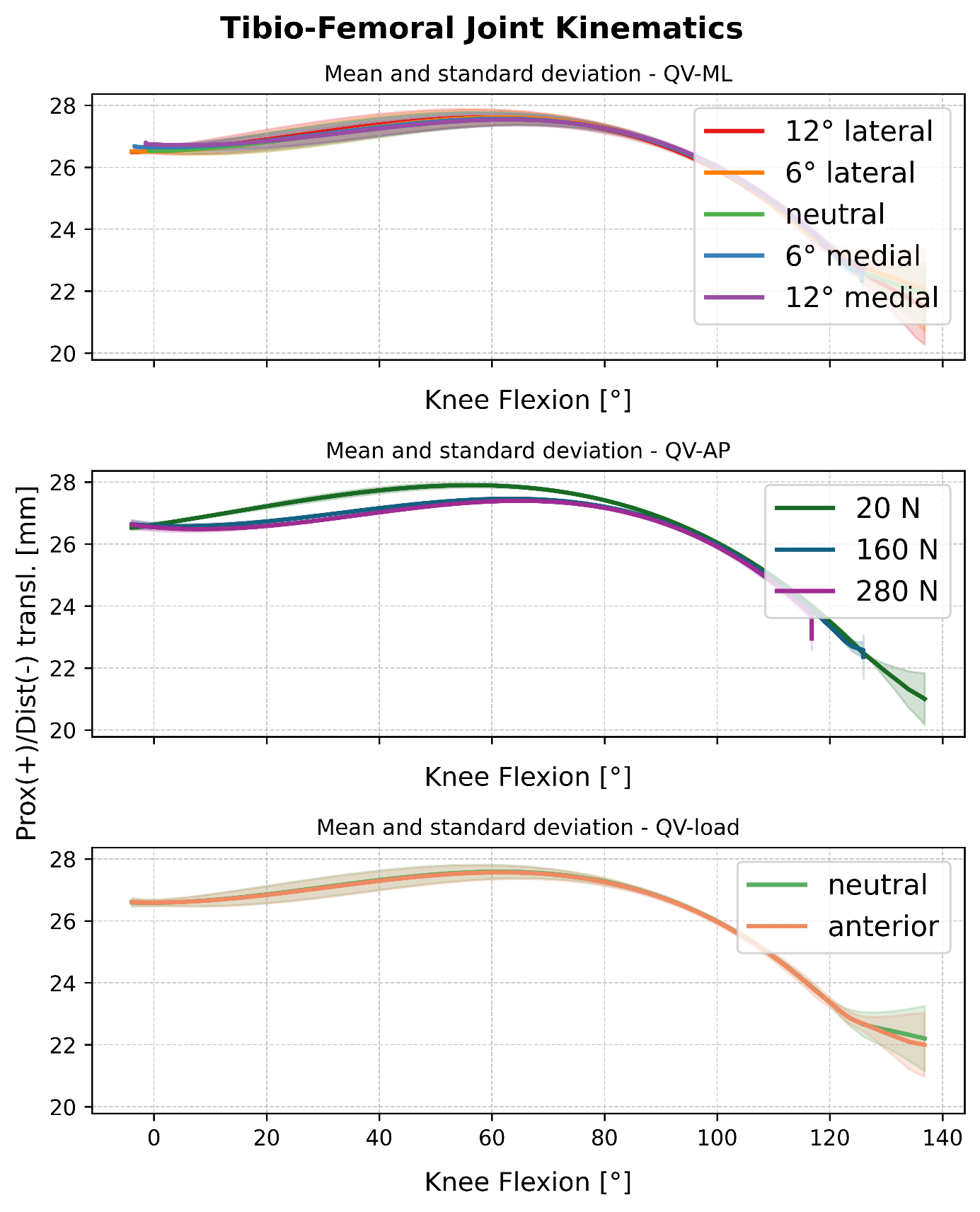
 Figure S4_5 Proximal/distal translation of one specimen (#1) with respect to the knee flexion for all the tests, grouped by QV-ML in the top, QV-load in the middle and QV-AP in the bottom


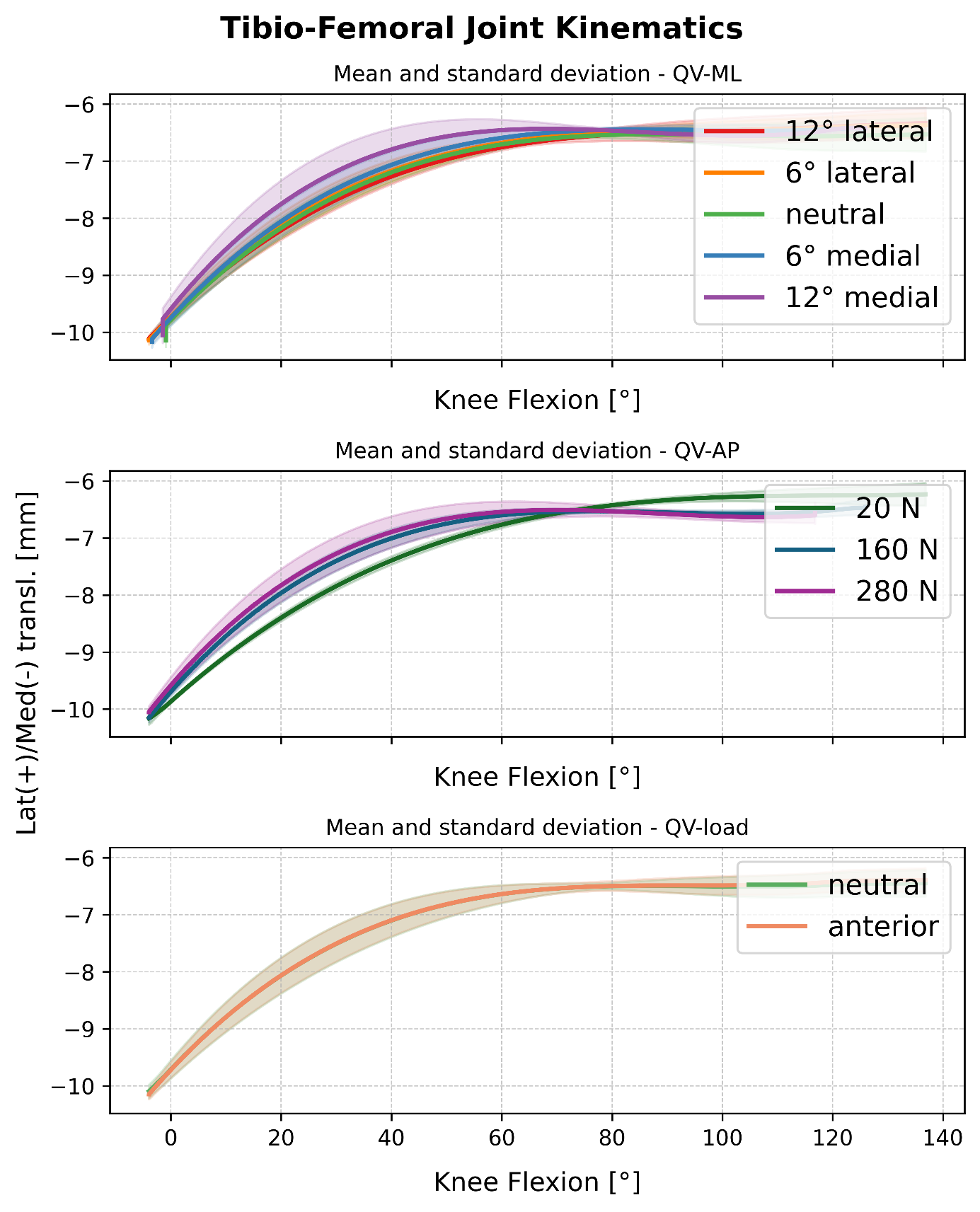
 Figure S4_6 Lateral/medial translation of one specimen (#1) with respect to the knee flexion for all the tests, grouped by QV-ML in the top, QV-load in the middle and QV-AP in the bottom


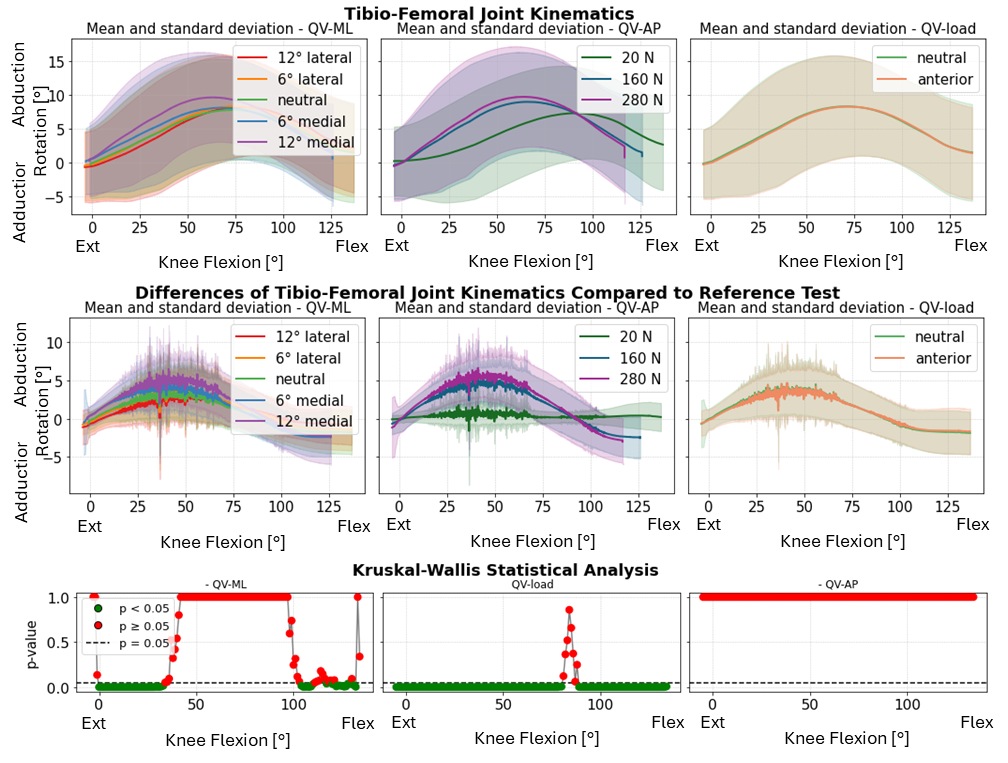


Figure S4_7: Abduction-Adduction rotation of the femur. TOP: absolute values as a function of knee flexion angle (the median and standard deviation between 12 specimens are plotted). CENTER: differences of all tests compared with the reference test (QVload = 20 N, QVML = neutral, QVAP = posterior). Left shows the difference as a function of QVML, middle QVload, right QVAP. BOTTOM: significance of the differences plotted at the center. The p-value trend is plotted for the three parameters (left QVML, middle QVload, right QVAP); the significant values are highlighted in green (p<0.05), the non significant ones in red (p≥0.05).


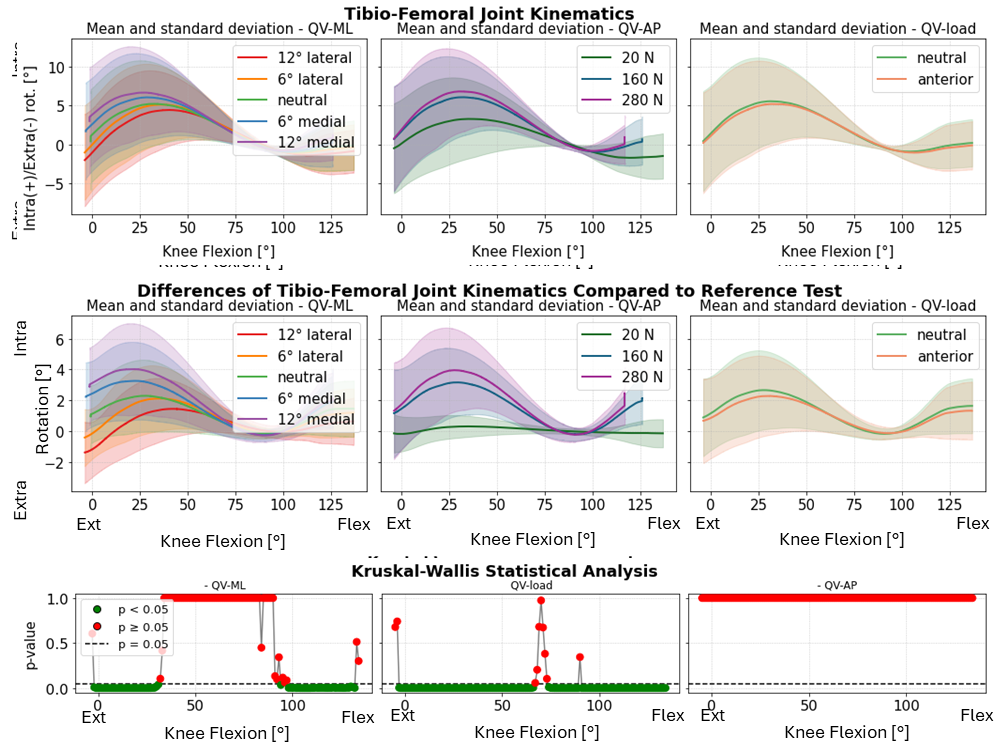


Figure S4_8: Intra-Extra rotation of the femur. TOP: absolute values as a function of knee flexion angle (the median and standard deviation between 12 specimens are plotted). CENTER: differences of all tests compared with the reference test (QVload = 20 N, QVML = neutral, QVAP = posterior). Left shows the difference as a function of QVML, middle QVload, right QVAP. BOTTOM: significance of the differences plotted at the center. The p-value trend is plotted for the three parameters (left QVML, middle QVload, right QVAP); the significant values are highlighted in green (p<0.05), the non significant ones in red (p≥0.05).


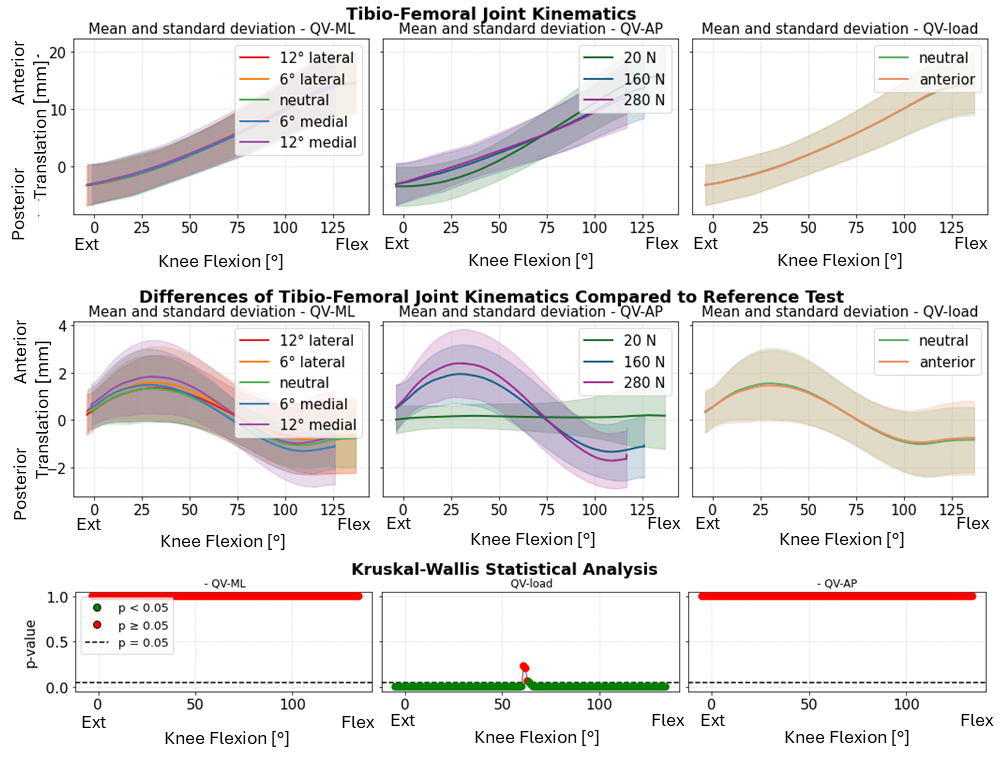


Figure S4_9: Anterior-Posterior translation of the femur. TOP: absolute values as a function of knee flexion angle (the median and standard deviation between 12 specimens are plotted). CENTER: differences of all tests compared with the reference test (QVload = 20 N, QVML = neutral, QVAP = posterior). Left shows the difference as a function of QVML, middle QVload, right QVAP. BOTTOM: significance of the differences plotted at the center. The p-value trend is plotted for the three parameters (left QVML, middle QVload, right QVAP); the significant values are highlighted in green (p<0.05), the non significant ones in red (p≥0.05).


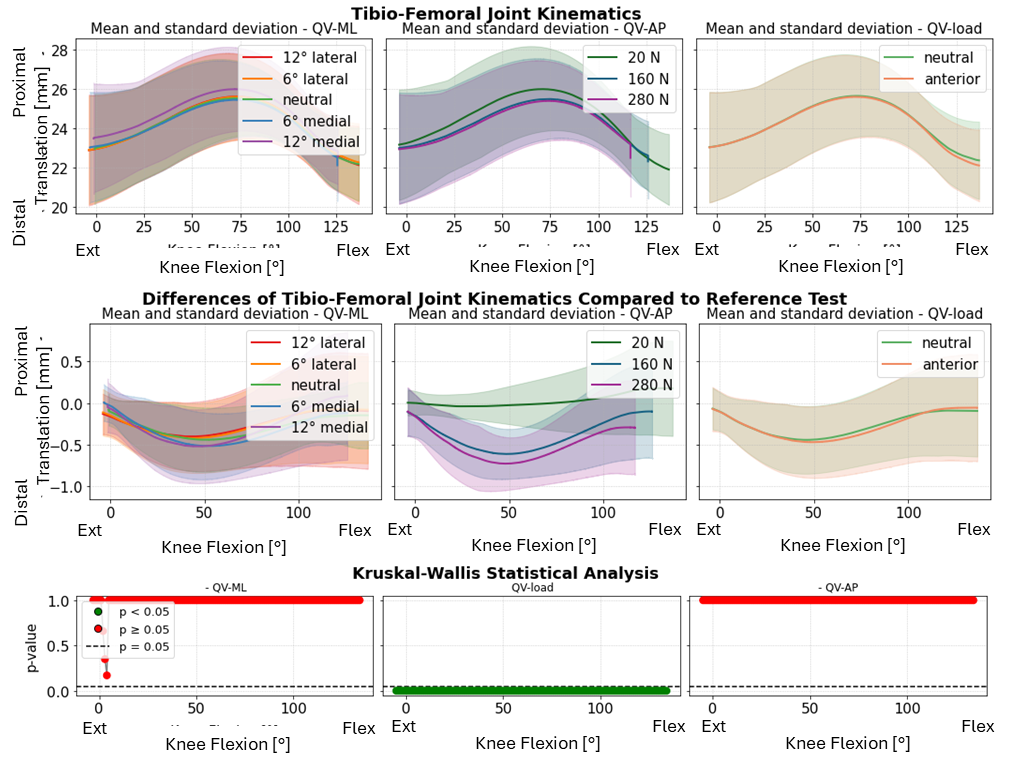


Figure S4_10: Proximal-Distal translation of the femur. TOP: absolute values as a function of knee flexion angle (the median and standard deviation between 12 specimens are plotted). CENTER: differences of all tests compared with the reference test (QVload = 20 N, QVML = neutral, QVAP = posterior). Left shows the difference as a function of QVML, middle QVload, right QVAP. BOTTOM: significance of the differences plotted at the center. The p-value trend is plotted for the three parameters (left QVML, middle QVload, right QVAP); the significant values are highlighted in green (p<0.05), the non significant ones in red (p≥0.05).green line.


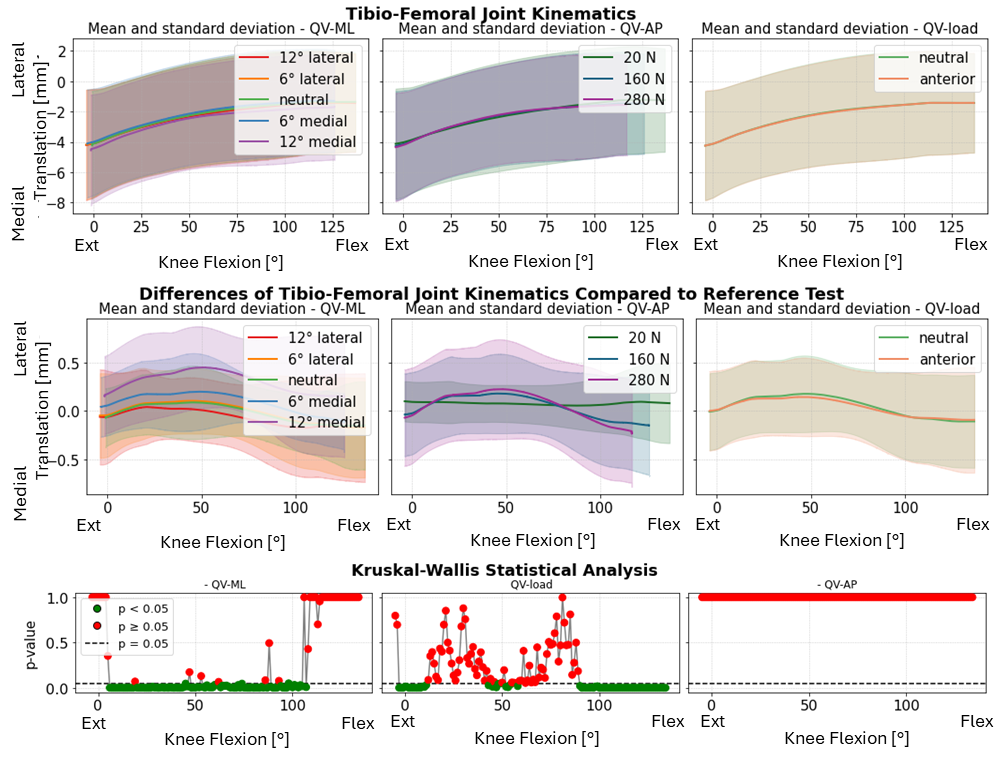


Figure S4_11: Medial-Lateral translation of the femur. TOP: absolute values as a function of knee flexion angle (the median and standard deviation between 12 specimens are plotted). CENTER: differences of all tests compared with the reference test (QVload = 20 N, QVML = neutral, QVAP = posterior). Left shows the difference as a function of QVML, middle QVload, right QVAP. BOTTOM: significance of the differences plotted at the center. The p-value trend is plotted for the three parameters (left QVML, middle QVload, right QVAP); the significant values are highlighted in green (p<0.05), the non significant ones in red (p≥0.05).

## **SUPPLEMENTARY MATERIALS #5**

### **Finite helical axes**

#### Introduction

In the main article the joints movement is presented using the Grood and Santay convention [2]. This convention can be seen as a specific implementation of the Euler angle method, where the rotation sequence and axes are carefully chosen to better represent knee kinematics. However, in literature, especially in the clinical field is very common to describe the joint movement using a series of finite helical axes. The Euler method describes motion through three rotations and three translations[4], which are dependent on the chosen coordinate system were among the first to use the helical axis method to describe knee joint motion. Nevertheless, with a generally accepted definition of the reference frame, rotation parameters provide a useful characterization of knee joint motion in anatomical terms [2].

Alternatively, the helical axis method describes motion by identifying a single axis about which both rotation and translation occur at each step [5], [6]. This approach makes the motion description independent of the chosen reference frame.

However, the helical axis method has some limitations. The axis is undefined in the case of pure translations, and its position and direction are highly sensitive to measurement errors when rotations are small. Moreover, for our specific analysis, we consider that helical axis visualization does not highlight certain differences as clearly as the Grood and Suntay convention. Nonetheless, it is widespread use to describe the tibiofemoral kinematic [7], [8], [9], while not so common for the patellofemoral joint [10]. Given its relevance in understanding joint motion, we provide this additional analysis for completeness, presenting it exclusively in the supplementary materials.

#### Methods

Using the data from specimen #1, obtained as described in the "Materials and Methods" section of the main article, we computed the relative position of the femur with respect to the tibia and of the patella with respect to the femur at 10° intervals of knee flexion. For each joint, we then calculated the transformation matrix between successive steps.

Applying Chasles’ theorem, we derived the helical axes of both the tibiofemoral and patellofemoral motion in 10° increments of knee flexion. The helical axes were computed across a flexion range of 0–120°. The helical axes here reported correspond to the test computed with QV-load=20N and QV-AP=neutral.

#### Results

##### Patellofemoral joint


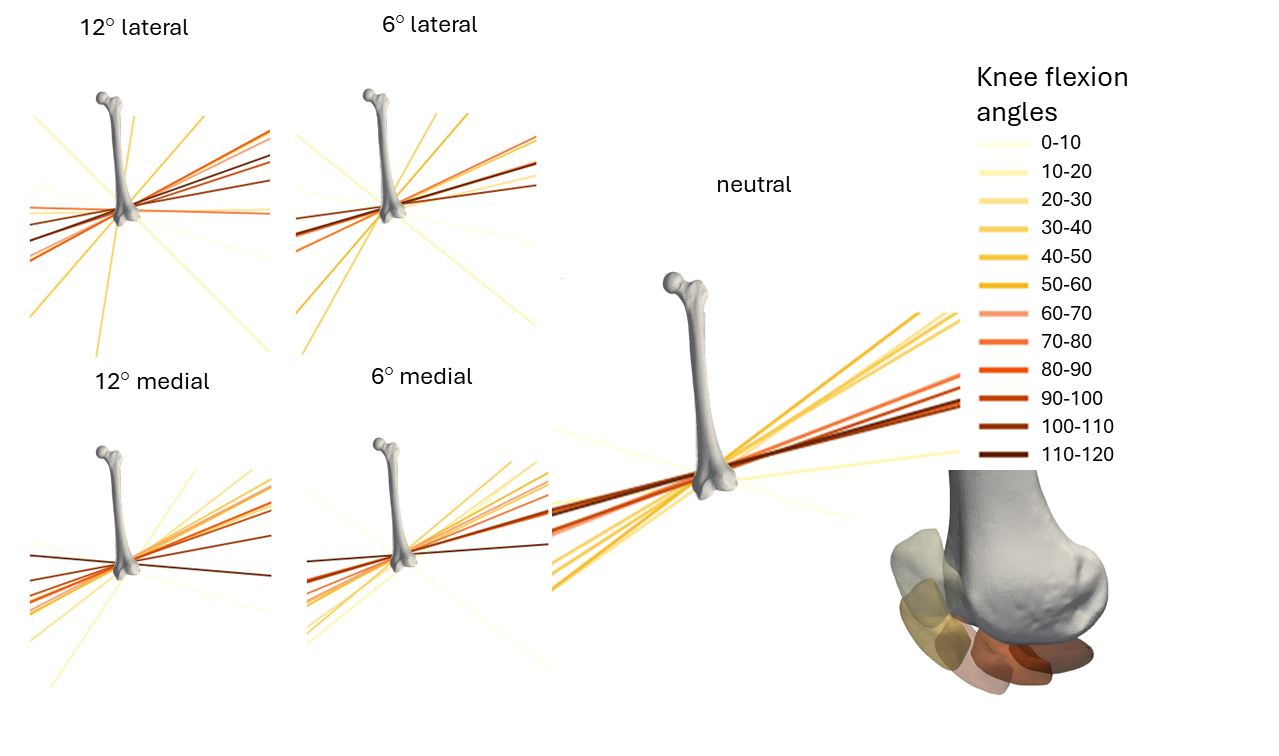
Figure S3_3 Helical axes of patellar motion relative to the femur for a single specimen under a quadriceps vector load (QV-load) of 20 N and an anteroposterior direction (QV-AP) set to neutral. Each subplot shows the helical axes corresponding to different mediolateral directions (QV-ML). With the shades of orange represented the knee flexion angles. The rightmost schematic representation illustrates the patellar movement in the neutral condition.
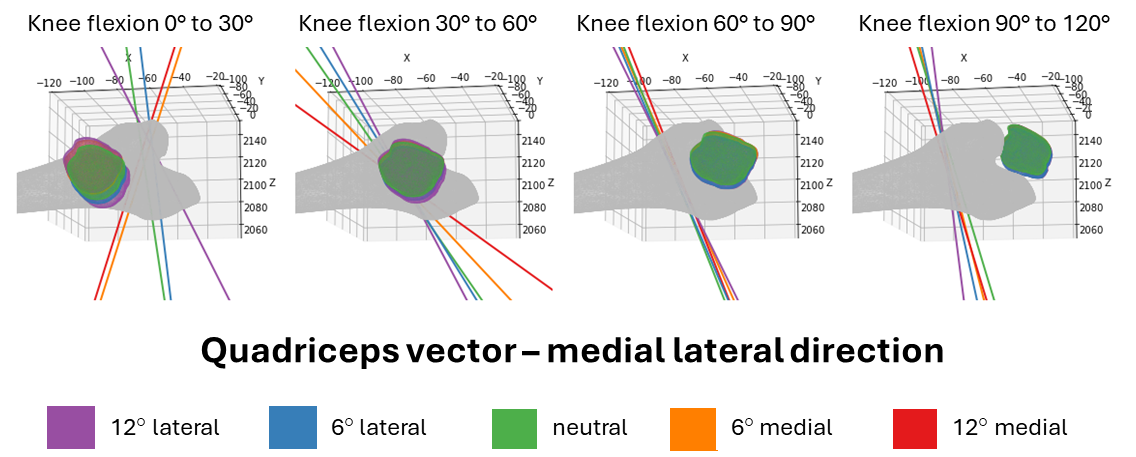


Figure S3_4 - Helical axes of patellar motion relative to the femur for a single specimen under a quadriceps vector load (QV-load) of 20 N and an anteroposterior direction (QV-AP) set to neutral. Each subplot represents a different knee flexion phase: early (0–30°), middle (30–60°), late (60–90°), and deep flexion (90–120°). The different colors correspond to distinct mediolateral directions of the quadriceps vector (QV-ML), as indicated in the legend.

##### Tibiofemoral joint


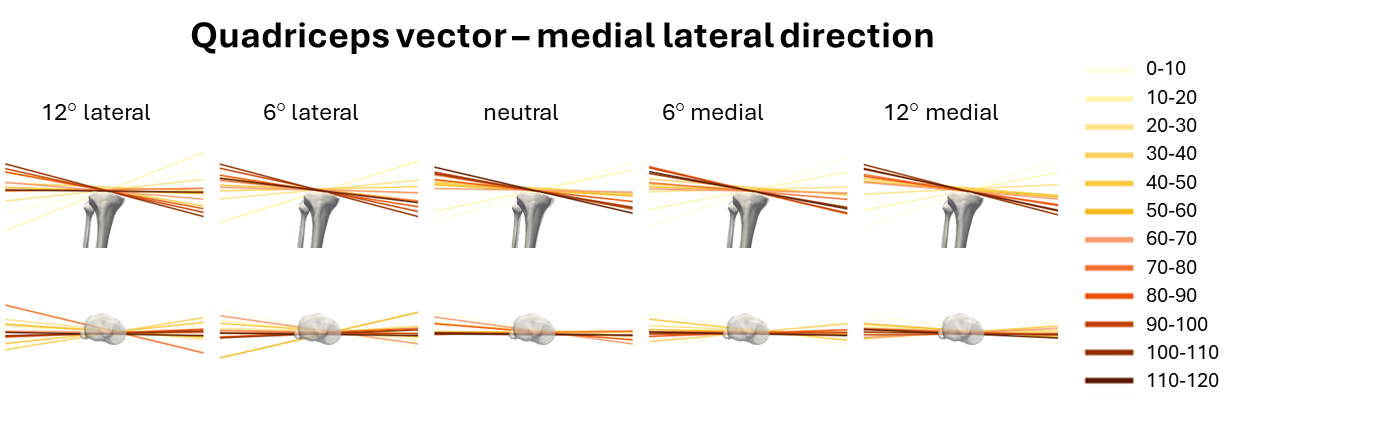


Figure S3_5 - Helical axes of femoral motion relative to the tibia for a single specimen under a quadriceps vector load (QV-load) of 20 N and an anteroposterior direction (QV-AP) set to neutral. Each subplot shows the helical axes corresponding to different mediolateral directions (QV-ML). With the shades of orange represented the knee flexion angles. In the top row a frontal view, in the bottom an axial view.


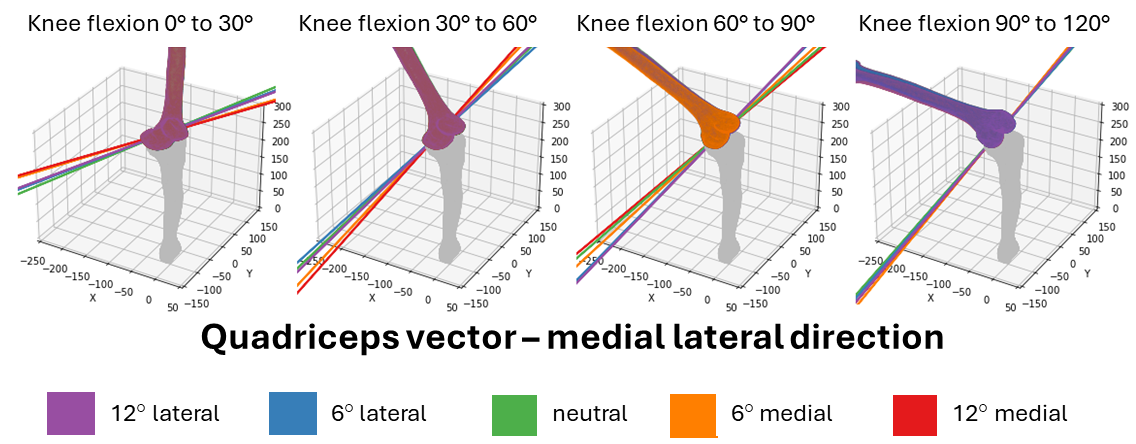


Figure S3_5 - Helical axes of femoral motion relative to the tibia for a single specimen under a quadriceps vector load (QV-load) of 20 N and an anteroposterior direction (QV-AP) set to neutral. Each subplot represents a different knee flexion phase: early (0–30°), middle (30–60°), late (60–90°), and deep flexion (90–120°). The different colors correspond to distinct mediolateral directions of the quadriceps vector (QV-ML), as indicated in the legend.

#### **References for the Supplementary Materials**

[1] W. J. Anderst and S. Tashman, “A method to estimate in vivo dynamic articular surface interaction,” *J. Biomech.*, vol. 36, no. 9, pp. 1291–1299, Sep. 2003, doi: 10.1016/S0021-9290(03)00157-X.

[2] E. S. Grood and W. J. Suntay, “A joint coordinate system for the clinical description of three-dimensional motions: application to the knee,” *J. Biomech. Eng.*, vol. 105, no. 2, pp. 136–144, May 1983, doi: 10.1115/1.3138397.

[3] A. A. Amis, W. Senavongse, and A. M. J. Bull, “Patellofemoral kinematics during knee flexion-extension: an in vitro study,” *J. Orthop. Res. Off. Publ. Orthop. Res. Soc.*, vol. 24, no. 12, pp. 2201–2211, Dec. 2006, doi: 10.1002/jor.20268.

[4] L. Blankevoort, R. Huiskes, and A. de Lange, “The envelope of passive knee joint motion,” *J. Biomech.*, vol. 21, no. 9, pp. 705–720, Jan. 1988, doi: 10.1016/0021-9290(88)90280-1.

[5] H. J. Woltring, R. Huiskes, A. De Lange, and F. E. Veldpaus, “Finite centroid and helical axis estimation from noisy landmark measurements in the study of human joint kinematics,” *J. Biomech.*, vol. 18, no. 5, pp. 379–389, Jan. 1985, doi: 10.1016/0021-9290(85)90293-3.

[6] L. Blankevoort, R. Huiskes, and A. de Lange, “Helical axes of passive knee joint motions,” *J. Biomech.*, vol. 23, no. 12, pp. 1219–1229, Jan. 1990, doi: 10.1016/0021-9290(90)90379-H.

[7] A. J. van den Bogert, C. Reinschmidt, and A. Lundberg, “Helical axes of skeletal knee joint motion during running,” *J. Biomech.*, vol. 41, no. 8, pp. 1632–1638, Jan. 2008, doi: 10.1016/j.jbiomech.2008.03.018.

[8] F. Temporiti *et al.*, “Dispersion of knee helical axes during walking in young and elderly healthy subjects,” *J. Biomech.*, vol. 109, p. 109944, Aug. 2020, doi: 10.1016/j.jbiomech.2020.109944.

[9] S. Konda *et al.*, “Comparison of finite helical axes of normal and anatomically designed prosthetic knees,” *Clin. Biomech.*, vol. 65, pp. 57–64, May 2019, doi: 10.1016/j.clinbiomech.2019.03.018.

[10] Z. Yu *et al.*, “Relationship between patellofemoral finite helical axis and femoral trans-epicondylar axis using a static magnetic resonance-based methodology,” *J. Orthop. Surg.*, vol. 16, no. 1, p. 212, Mar. 2021, doi: 10.1186/s13018-021-02328-2.
